# Pools of Independently Cycling Inositol Phosphates Revealed by Pulse Labeling with ^18^O‑Water

**DOI:** 10.1021/jacs.4c16206

**Published:** 2025-05-15

**Authors:** Geun-Don Kim, Guizhen Liu, Danye Qiu, Maria Giovanna De Leo, Navin Gopaldass, Jacques Hermes, Jens Timmer, Adolfo Saiardi, Andreas Mayer, Henning Jacob Jessen

**Affiliations:** † Département d’immunobiologie, 27213 Université de Lausanne, CH-1066 Epalinges, Switzerland; ‡ Institute of Organic Chemistry, 9174 University of Freiburg, 79104 Freiburg, Germany; § CIBSS—Centre for Integrative Biological Signaling Studies, University of Freiburg, 79104 Freiburg, Germany; ∥ Institute of Physics, University of Freiburg, 79104 Freiburg, Germany; ⊥ Medical Research Council, Laboratory for Molecular Cell Biology, University College London, WC1E 6BT London, U.K.

## Abstract

Inositol phosphates control many central processes in eukaryotic cells including nutrient availability, growth, and motility. Kinetic resolution of a key modulator of their signaling functions, the turnover of the phosphate groups on the inositol ring, has been hampered by slow uptake, high dilution, and constraining growth conditions in radioactive pulse-labeling approaches. Here, we demonstrate a rapid (seconds to minutes) and nonradioactive labeling strategy of inositol polyphosphates through ^18^O-water in yeast, human cells, and amoeba, which can be applied in any media. In combination with capillary electrophoresis and mass spectrometry, ^18^O-water labeling simultaneously dissects the in vivo phosphate group dynamics of a broad spectrum of even rare inositol phosphates. The good temporal resolution allowed us to discover vigorous phosphate group exchanges in some inositol polyphosphates and pyrophosphates, whereas others remain remarkably inert. We propose a model in which the biosynthetic pathway of inositol polyphosphates and pyrophosphates is organized in distinct, kinetically separated pools. While transfer of compounds between those pools is slow, each pool undergoes rapid internal phosphate cycling. This might enable the pools to perform distinct signaling functions while being metabolically connected.

## Graphical abstract

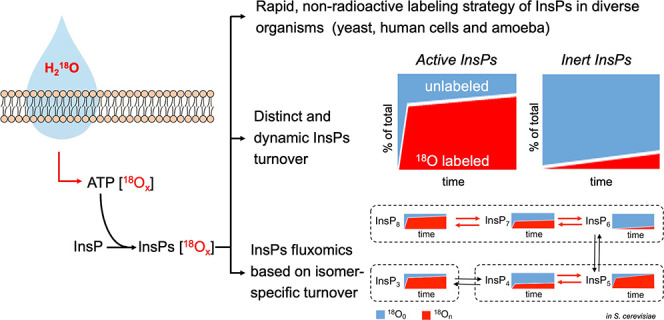

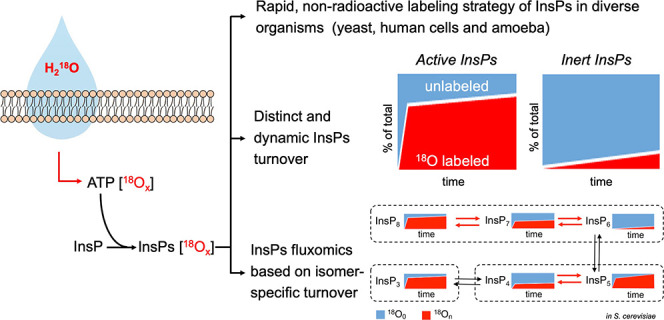

## 1 Introduction

Water-soluble cytosolic inositol phosphates (InsPs) can be synthesized by rearrangement of glucose-6-phosphate to inositol-3-phosphate or emanate from the hydrolysis of inositol lipids (Figure 1). Inositol-containing metabolites have key signaling roles in eukaryotic cells. Phosphatidylinositol lipids (PtdInsPs or simply PIPs) determine the identity of organelles and organize vesicular traffic between them.^1^ The biologically occurring species with phosphate groups in different positions of the inositol headgroup are important to cell polarity, movement and mechanotransduction, the release of growth factors, neurotransmitters, and hormones.^2^ The cytosolic or water-soluble InsPs have equally diverse and important signaling functions, including DNA stability, RNA transport, transcriptional control, MAP kinase signaling, maintaining energy homeostasis, and many others.^3–5^ Inositol pyrophosphates (PP-InsPs), the most highly phosphorylated water-soluble InsPs, which contain diphosphate groups, link phosphate homeostasis to adenosine triphosphate (ATP) levels across species, in part by signaling via SPX (Syg1/Pho81/Xpr1) domain-containing proteins.^6–12^

**Figure 1:**
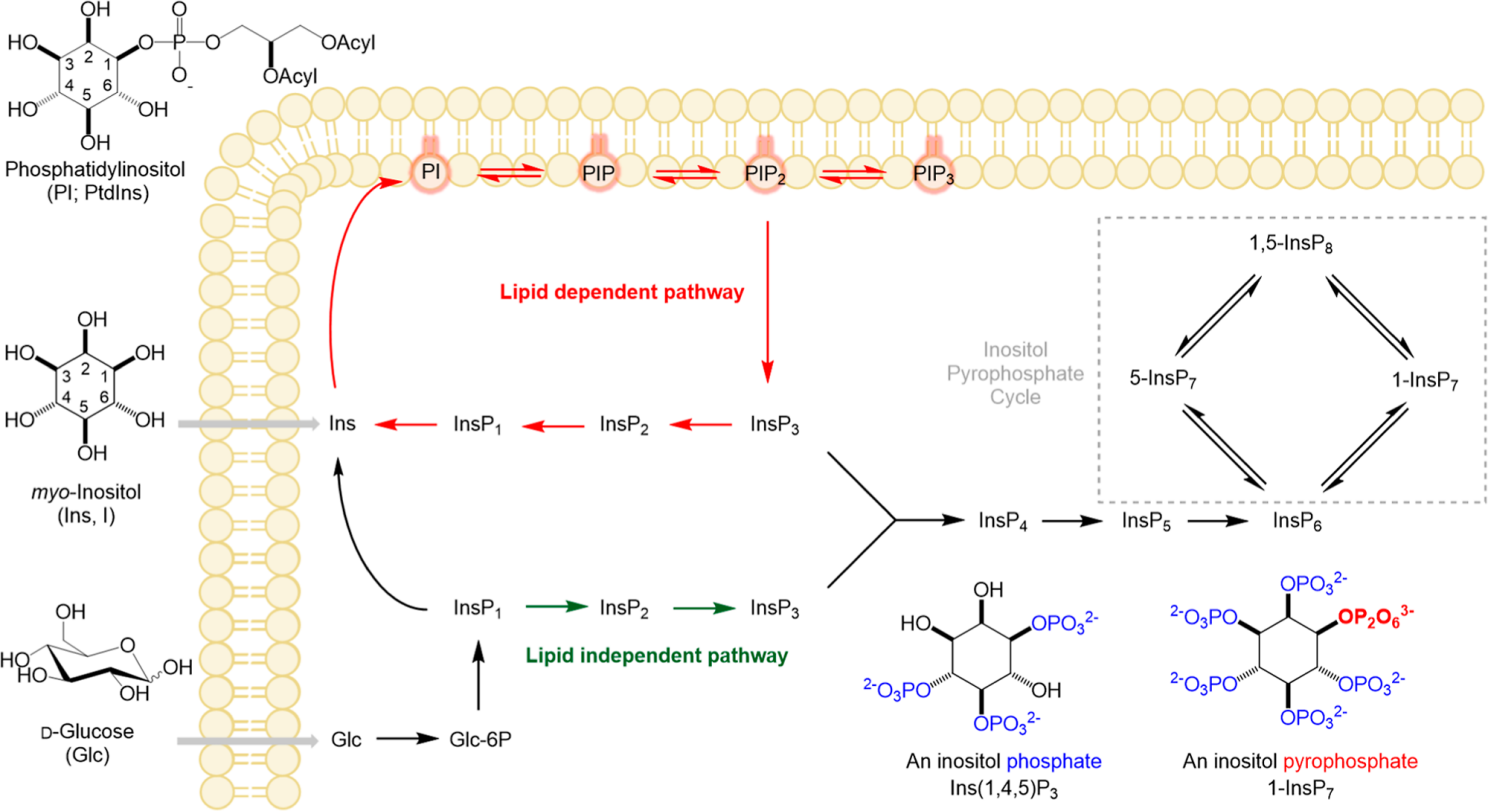
Pathways for synthesis of lipidic and generic soluble inositol phosphates. Inositol can either be taken up by cells or synthesized from glucose after uptake and phosphorylation. Cytosolic inositol phosphates are generated from PtdInsP_2_ (PIP2) through phospholipase C (PLC), generating diacylglycerol and InsP_3_.^20^ In mammalian cells, an additional lipid-independent pathway can generate InsP_3_ directly from glucose.^18^ Forward phosphorylation by kinases up to InsP_8_ is possible while phosphatases remove the phosphate groups, generating lower phosphorylated forms. The balance between kinases and phosphatases will likely occur at different rates controlled by metabolic and/or signaling inputs. Therefore, we can envisage an InsP network constantly turning over, harmonizing the steady-state concentrations of the different metabolites.

In many of the processes mentioned above, InsPs and PtdInsPs are interconverted, leading to alterations of the number and/or the positioning of their phosphate groups. Measuring the steady-state levels of InsPs and PP-InsPs has usually been achieved by strong anion exchange-high-performance liquid chromatography (SAX-HPLC) after metabolic labeling with ^3^H or ^14^C-inositol, but this is a lengthy process that requires 24 h in yeast to several days in mammalian cells under constraining growth conditions.^13^ More recently, capillary electrophoresis-mass spectrometry (CE-MS) has been introduced to monitor absolute InsP and PP-InsP levels,^14–17^ and stable isotope ^13^C labeling with glucose and inositol has revealed alternative pathways of soluble InsP synthesis from membrane lipids or glucose (Figure 1).^15,18^ Although these approaches can be used to separate and quantify the steady-state levels of InsPs and PP-InsPs, the fast dynamics of the interconversion of the phosphate groups in cells has remained difficult to capture. This is in large part due to inherent limitations and complexity of the pulse-labeling approaches with radiolabeled tracers, which have so far been necessary for such analyses.^19^

Inositol phosphates can be labeled through feeding the cells with radiolabeled inositol or by adding isotope-labeled phosphate.^13^ Inositol uptake is slow, and labeling is usually performed to isotopic equilibrium, involving a culture in inositol-depleted media over several generations. Moreover, inositol can be synthesized by cells from glucose, which leads to unlabeled inositol that cannot be traced.^14,18^ ^32^P labeled inositol phosphate enables us to study the sequential addition of phosphate but is limited because even after 40 min of labeling, ATP does not reach isotopic equilibrium.^21–23^ Phosphate labeling is also comparatively slow because it relies on uptake through plasma membrane transporters. It is subjected to isotopic dilution both in the medium, where phosphate is an essential macronutrient, and in the cell, which has cytosolic phosphate concentrations of up to 20 mM^24^ and storage pools for phosphate. Furthermore, P_i_ utilization, and thereby the ability to label the internal P_i_ pool through P_i_ uptake, is strongly influenced by biosynthesis and cell growth, which change as a function of the culture conditions.

While steady-state levels of metabolites themselves are an important information, the dynamic turnover of metabolites in this steady state, the metabolic flux, reflects the activity of a metabolic pathway.^25^ Flux provides additional information about the metabolic state and can constitute a signal for the cell.^26–28^ It can help us understand how a steady-state concentration is maintained and how quickly a system can adjust to perturbations.^18,29,30^ For example, the use of NaF as metabolic trap to block metabolic fluxes depending on phosphatases revealed dynamic phosphorylation of the abundant InsP_6_ (50–100 μM) into scarce PP-InsPs (0.1–2 μM), with a turnover of ca. 50% of the whole InsP_6_ pool within 1 h.^31^

Measuring the dynamic turnover of phosphorylated metabolites should ideally rely on direct labeling of the phosphate groups. This can be achieved by using isotopes. For phosphorus, only radioactive nuclides are available. Furthermore, phosphorus can be absorbed by cells only in the form of organic or inorganic phosphate (P_i_). The uptake process can be quite slow, rendering it difficult to monitor rapid processes, as, for example, even after 40 min radioactive P_i_ treatment of duckweed, ATP is not labeled to equilibrium.^22^ Another option of labeling the phosphate group is by stable isotope labeling using ^18^O-enriched water.^30,32–35^ Water enters cells within seconds. It is incorporated into P_i_ by hydrolytic enzymatic reactions. A purely chemical exchange of P_i_ is inefficient.^30,36^ Hydrolysis occurs vigorously on cellular nucleotides, as exemplified by ATP, for which the entire cellular pool can turn over in seconds.^30,37^ This rapid cycling entrains a similarly rapid introduction of ^18^O into the hydrolytic product, P_i_.

The incorporation of ^18^O into ATP (9 oxygens are exchangeable via different enzymatic mechanisms) has been studied extensively using direct ^31^P nuclear magnetic resonance (NMR) approaches and liquid chromatography–mass spectrometry, as well as by an indirect gas chromatography–mass spectrometry approach.^25,38^ Due to the inherently low sensitivity of NMR, this approach will not be effective to monitor scarce signaling molecules with high turnover rates. Of particular interest for phosphotransfer reactions is the appearance of the isotope label in the γ-phosphate of ATP as this is the most readily transferable phosphate group. In rat hearts, for example, the γ-phosphate in every ATP is turned over 34 times per minute, the β-phosphate 12 times, and the α-phosphate only once a minute.^25^ ATP-synthase can incorporate ^18^O from ^18^O-water even multiple times into ATP before releasing its product into the cell.^30,39^ These studies have consistently demonstrated very rapid incorporation of ^18^O labels, particularly in the γ-phosphate. Earlier experiments with ^18^O-water have also underpinned the existence of different compartmentalized ATP pools (metabolic and nonmetabolic), which vary with cell types and physiological states.^30^ While ^18^O-labeling was exploited for a limited number of analyses of ATP turnover and labeled synthetic ^18^O ATP has been used in phosphoproteomics,^40,41^ the potential of an extension of this concept to other areas of phosphorylated metabolites received only limited attention. Recently, a study has shown incorporation of ^18^O labels into the PtdInsPs phosphate diester after incubation of mammalian cells with ^18^O-water.^34,42^ Furthermore, the advent of new chemical synthesis approaches made ^18^O labeled reference compounds available, enabling ionization and fragmentation studies to improve assignments and quantifications of inositol phosphates.^43–45^

Here, we use pulse-labeling to explore the turnover of inositol polyphosphates of yeast, amoeba, and mammalian cells. Since these are in large part low-abundance metabolites (nanomolar to low micromolar concentration), we rely on the sensitivity and separation power of capillary electrophoresis coupled to both triple quadrupole (QQQ) and quadrupole time-of-flight (qTOF) mass spectrometry for their analysis, of which the first one is more sensitive. Our in vivo approach is based on the rapid permeation of ^18^O water into cells and on the extremely rapid turnover of the cellular P_i_ through nucleotide hydrolysis.^37,46^ Our results provide an entry point into coupled InsP and PP-InsP fluxomics, revealing unexpectedly high turnover rates of some metabolites, metabolic lethargy of others, selective impact of applied conditions/knockouts on ^18^O labeling kinetics, and, most notably, the existence of several independently cycling InsP pools (workflow shown in Figure 2).

**Figure 2:**
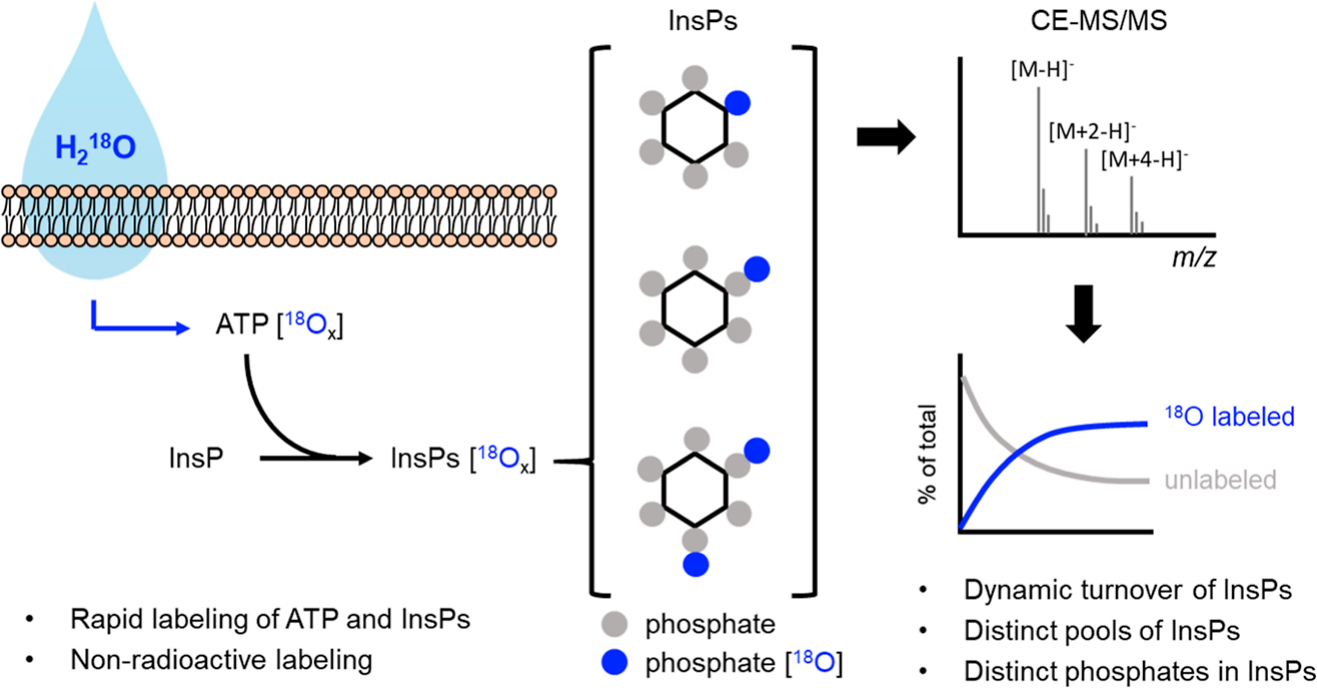
Workflow for the analysis of cellular ^18^O labeled ATP and InsP pools through ^18^O-water labeling, combined with CE-MS analysis.

## 2 Results

### 2.1 Rapid ^18^O Labeling of ATP in Yeast

To label yeast cells, cultures that were logarithmically growing in SC (synthetic complete) medium were transferred to the same SC medium made with 50% ^18^O-water. 50% ^18^O-water suffices to label the entire pool of P_i_ almost quantitatively because, in isotopic equilibrium, the probability of P_i_ remaining with four unlabeled oxygen atoms is equal to 1/16 (6%). In this medium, P_i_ is the sole source of phosphate. At different time points, aliquots of the culture were extracted with perchloric acid,^47,48^ and the extracts were analyzed by CE-MS (Figure 2). The number of labels was assigned by combination of internal standards and high-resolution qTOF mass spectrometry (Figure 3A). Already at the first time point taken, after 1 min, ATP with one-four ^18^O atoms was detected (Figure 3A). Labeling proceeded rapidly, even at 20 °C, reaching up to seven ^18^O labels after 60 min. The ^18^O labels also rapidly penetrated the InsPs, which could be analyzed from the same extracts used for nucleotide analytics. The gradual incorporation of multiple labels poses a problem for rare analytes because the already low abundance of ions is now distributed over all isotopologues. This renders the MS analysis of rare analytes challenging, such as InsP_5_ and inositol pyrophosphates (InsP_7_ and InsP_8_), and complicates the detection of analytes with a large number of ^18^O atoms. To try to quantify all these rare analytes, CE-MS with a triple quadrupole system (CE-QQQ) in multiple reaction monitoring (MRM) mode was employed because it offers higher sensitivity than the qTOF system.

**Figure 3:**
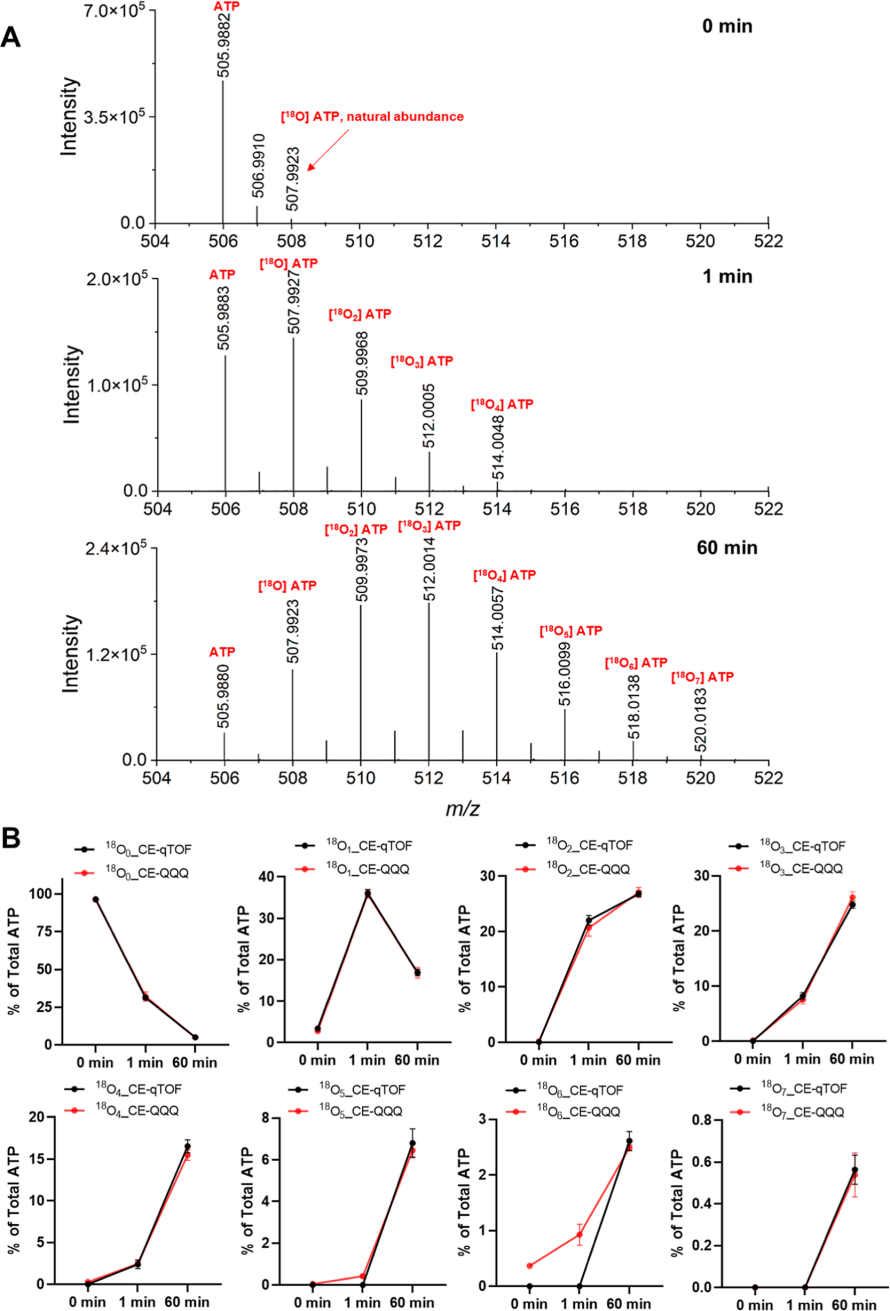
(A) qTOF MS analysis of ATP. The analysis reveals the kinetics of ^18^O incorporation into ATP at different exchangeable positions. Wild-type yeast cells were grown in SC medium at 20 °C to slow down the incorporation. At the 0 min time point, the medium was changed to SC medium prepared with 50% ^18^O-water. Samples were harvested at different time points (1 and 60 min), extracted with perchloric acid and TiO_2_, and analyzed by qTOF mass spectrometry. Theoretical [M-H]^−^ for ATP, [^18^O] ATP, [^18^O_2_] ATP, [^18^O_3_] ATP, [^18^O_4_] ATP, [^18^O_5_] ATP, [^18^O_6_] ATP, and [^18^O_7_] ATP are 505.9885, 507.9927, 509.9970, 512.0012, 514.0055, 516.0097, 518.0139, and 520.0182, respectively. (B) The incorporation of ^18^O into ATP was analyzed at 0, 1, and 60 min using CE-qTOF (MS1) and CE-QQQ (MRM) methods. The means of 3 samples with standard deviation are shown.

Specified transitions from precursor to product ions were optimized by the MassHunter optimizer with injection of standards to maximize the product ion signal. The ion at m/*z* 408.0117, one of the major fragmented ions found corresponding to [M – H–H_3_PO_4_]^−^ for unlabeled ATP, was chosen as the product ion (Figure S1). An unexpected oxygen exchange (scrambling) on ATP was detected through analysis of the synthetic control compound γ-^18^O_2_-ATP^44^—which carries two defined ^18^O as nonbridging oxygens of the γ-phosphate (Figure S2) with >99% ^18^O_2_/^16^O_2_ ratio. However, its MS2 fragmentation showed partial scrambling of ^18^O in the gas phase under these conditions, i.e., we observed monolabeled γ-phosphate and monolabeled adenosine diphosphate (ADP). 49% of single ^18^O scrambles and 16% of double ^18^O scrambles were observed (Figure S2). It is mechanistically conceivable that such an exchange of oxygens might occur through a reversible ping-pong gas-phase reaction involving metaphosphates.

Scrambling was also observed when synthetic [^18^O_2_] 5-InsP_7_ was employed,^43^ which was only labeled on the nonbridging oxygens in the β-phosphate (Supplementary Figure S3). Optimization of the MS parameters with synthetic γ-^18^O_2_-ATP and [^18^O_2_] 5-InsP_7_ did not reduce the observed scrambling. Therefore, to accurately count the numbers of ^18^O labels in the analytes and avoid an underestimation of the extent of labeling, scrambled product ions had to be considered when using the MRM transitions by CE-QQQ. For example, the observed scrambled product ions, such as (^18^O_2_ ATP-H–H_3_PO_2_^18^O_2_)^−^, (^18^O_2_ ATP-H–H_3_PO_3_^18^O)^−^, and (^18^O_2_ ATP-H–H_3_PO_4_)^−^, were summed to calculate the total ^18^O_2_ ATP (Table S3). We also considered the scrambled product ions for InsPs. For example, the observed scrambled product ions (^18^O_2_ InsP_7_-2H–H_3_PO_4_)^2–^, (^18^O_2_ InsP_7_-2H–H_3_PO_3_^18^O)^2–^, and (^18^O_2_ InsP_7_-2H–H_3_PO_2_^18^O_2_)^2–^ were recorded with the same precursor ion, ^18^O_2_ InsP_7_ (Table S4). The MRM transitions for all analytes are detailed in Tables S3–S8. To assess whether our consideration of all scrambled product ions in the CE-QQQ method allows for accurate measurements of ^18^O incorporation ratios, we compared the quantitative results at 0, 1, and 60 min obtained using CE-qTOF (MS1 level, where no scrambling occurs) and CE-QQQ (MRM, accounting for all scrambled product ions). As shown in Figure 3B, there is excellent agreement between the results from CE-qTOF and CE-QQQ, except for ^18^O_6_ ATP. The slight deviation (less than 1%) observed at 1 min for ^18^O_6_ incorporation is due to the lower sensitivity of CE-qTOF in detecting ^18^O_6_ ATP at 1 min, whereas CE-QQQ demonstrates a higher sensitivity for the detection. This shows that our CE-QQQ method accurately and sensitively reveals ^18^O incorporation. In summary, using synthetic ^18^O labeled ATP and InsP_7_ allowed us to identify the scrambling of ^18^O during the MS/MS fragmentation in the gas phase and to optimize the MRM transitions and accurately count the numbers of ^18^O labels on ATP and InsPs by CE-QQQ.

### 2.2 Rapid Pulse-Labeling Resolves Separate, Cycling Pools of Soluble Inositol Polyphosphates in Yeast

Using our previously described CE-MS method,^14^ we monitored the time course of ^18^O incorporation from ^18^O-water into ATP by CE-QQQ. Already after 1 min at 20 °C more than 68% of the ATP pool was labeled (Figure 4A, for a representative example of extracted ion electropherograms see Figure S4). The ATP labeling tended to be even faster and was shifted to larger numbers of total ^18^O incorporation when 100% ^18^O-water was used in the culture medium (Figure 4B), which was, however, avoided for cost reasons.

**Figure 4:**
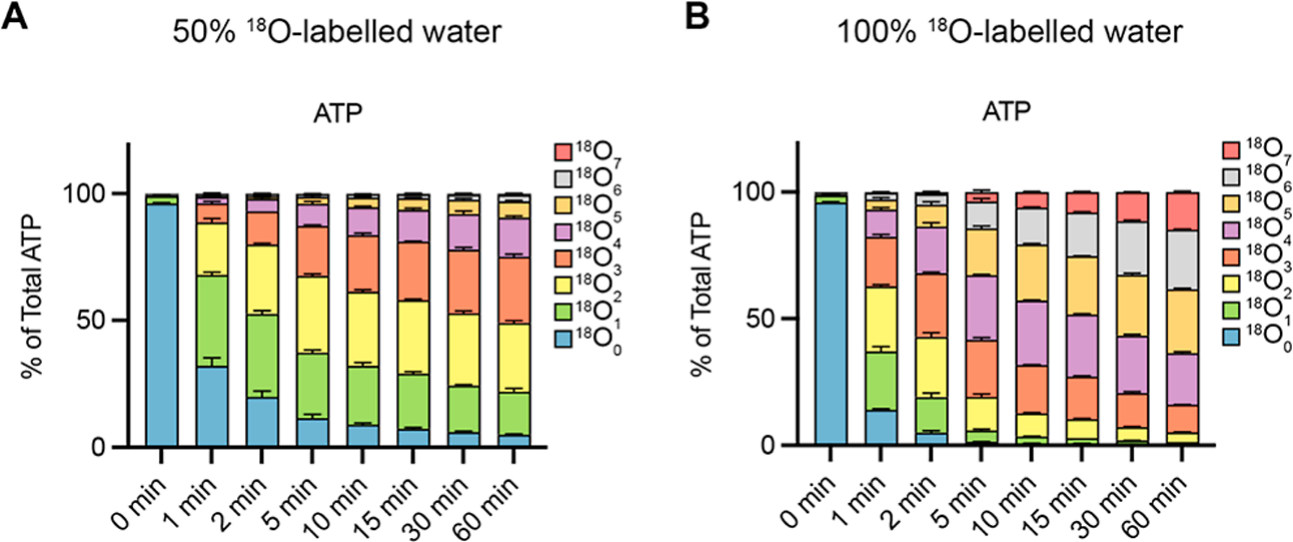
Kinetics of ^18^O entry into ATP of yeast under steady-state conditions. Time-dependent formation of ATP isotopologues in yeast with different numbers of ^18^O atoms studied by CE-QQQ with wide mass resolution. Wild-type yeast cells were grown logarithmically in SC medium at 20 °C. At the 0 min time point, the medium was changed to SC medium prepared with A 50% or B 100% of ^18^O-labeled water (99% enrichment). After further incubation at 20 °C for the indicated periods of time, cells were extracted with perchloric acid and TiO_2_. The graphs show the fractions of ATP that carried the indicated numbers of ^18^O at any position. The means of 3 samples with standard deviation are shown.

ATP does not undergo spontaneous ^16/18^oxygen exchange in ^18^O-water. Further addition of inositol hexakisphosphate kinase (IP6K1), which could potentially catalyze an exchange, did not have any effect either (Figure S5).

The ^18^O labels rapidly appeared in InsPs and PP-InsPs, which could be analyzed from the same extracts as used for ATP analytics by CE-MS, although measuring low-abundance metabolites (e.g., 5-InsP_7_ and 1,5-InsP_8_) has challenges due to mass resolution constraints and isotopic interference. These ^18^O labels result neither from spontaneous ^16/18^oxygen exchange in the presence of ^18^O-water (Figure S6), nor from extraction of InsPs and PP-InsPs (Figure S7). They are also not transferred by inositol hexakisphosphate kinase in the absence of ^18^O-ATP (Figures S8 and S9). Our measurements yielded ^18^O signals in InsP_7_ and InsP_8_ that were already above the expected natural abundance of ^18^O at the 0 min time point. Since the isotope frequency of ^18^O is 0.2% and inositol polyphosphates are very oxygen-rich molecules (e.g., 27 oxygens for InsP_7_), this leads to an expected fraction of ca. 5–6% of these compounds carrying at least one ^18^O. Our analytical protocol overestimates (from ca. 5% to 15% at the 0 min time point) this expected value only for InsP_7_ and InsP_8_, due to overlap with isotopic peaks of unlabeled PP-InsPs when using the wide mass resolution (full-width at half-maximum of 1.2 Da, ±0.6 Da mass error) of the quadrupole. We had to choose wide mass resolution in these experiments to attain the sensitivity needed to quantify ^18^O labeled InsP_7_ and InsP_8_, which are very low-abundance metabolites. Wide mass resolution can separate major isotopic clusters, such as carbon-based compounds dominated by ^12^C and ^13^C, referred to as M and M+1. In the case of InsP_7_ and InsP_8_, doubly charged precursor ions and product ions were detected, resulting in a skewing of the expected value from 5 to 15% at the 0 min time point. By contrast, the example of ATP representing analytes with singly charged precursor or product ions showed an excellent agreement between the results of unit mass resolution and wide mass resolution. This indicates that almost no skewing in the measurement of singly charged precursor or product ions occurs (Figures S10 and S11).

The skewing in InsP_7_ and InsP_8_ quantification could potentially be corrected by performing unit mass resolution measurements, which is specified to have only ±0.35 Da mass error (Figure S12). However, unit mass resolution, which results in an approximate 3-fold reduction of the signal intensity compared to wide mass resolution, was not sufficiently sensitive to quantify ^18^O labeled InsP_8_. With this information, we reanalyzed ^18^O labeled InsP_7_ in unit-mass resolution (Figures 5B and S17B) under optimal conditions (first-class capillary cut, freshly prepared sheath liquid, and fresh background electrolyte).^49^ This approach still did not enable the measurement of InsP_8_, which is therefore reported in wide mass resolution with the expected skewing (Figures 5A and S17A).

**Figure 5:**
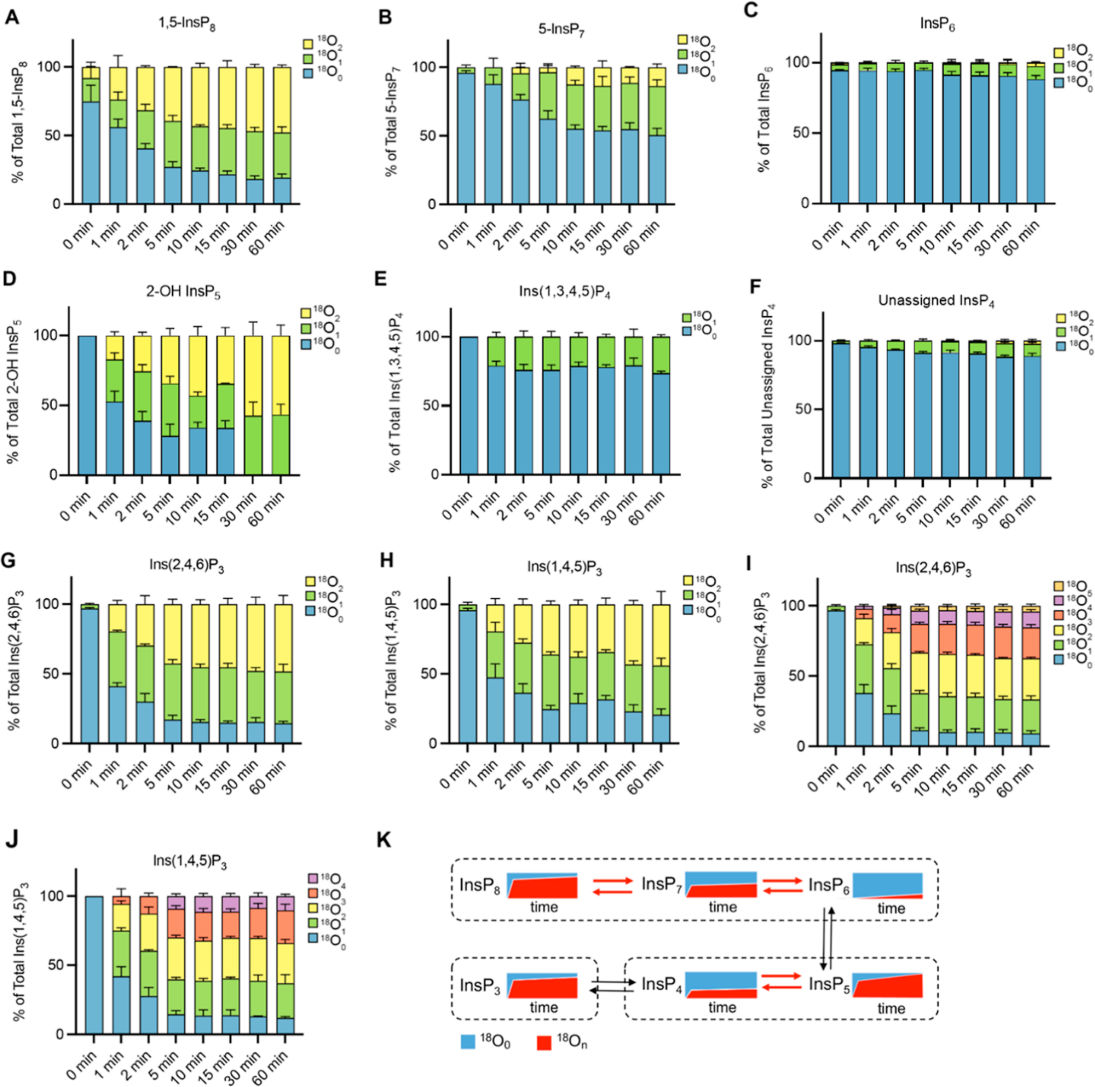
Kinetics of ^18^O entry into soluble InsPs of yeast under steady-state conditions. Wild-type yeast cells were grown logarithmically in SC medium at 20 °C. The medium was changed to SC medium prepared with 50% of ^18^O-labeled water. After further incubation at 20 °C for the indicated periods of time, cells were extracted with perchloric acid and TiO_2_ and analyzed by CE-QQQ or qTOF. Note that the levels of 1-InsP_7_ in these samples were too low to be reliably detected. The means of 3 samples with standard deviation is shown, representing (A) 1,5-InsP_8_, (B) 5-InsP_7_, (C) InsP_6_, (D) 2-OH-InsP_5_, (E) Ins­(1,3,4,5,)­P_4_, (F) unassigned InsP_4_, (G) Ins­(2,4,6)­P_3_, (H) Ins­(1,4,5)­P_3_, (I,J) higher numbers of ^18^O incorporation into InsP_3_ were observed by CE-qTOF of samples as in (G,H,K). Proposed pathways of interconversion. Rapid interconversion of metabolites is indicated by red arrows, and slow interconversion by black arrows. CE-QQQ with wide mass resolution was used in (A,C,D,E,F,G,H), and CE-QQQ with unit mass resolution was used in (B).

To estimate whether the described skewing effect changed its magnitude during the time course, mixtures of synthetic ^18^O_2_ 5-InsP_7_ and unlabeled 5-InsP_7_ with different compositions were prepared and analyzed by CE-QQQ using wide mass resolution. Raising the concentration of the ^18^O_2_ 5-InsP_7_ reduces the skewing of ^18^O 5-InsP_7_ from 10% to less than 3% (Figure S13). Also, in our time course experiments, the effect is largest at the zero time point (ca. 10–20%) and declines during increasing incorporation of ^18^O labels (Figure S15), which made a simple subtraction to the expected zero-time point value problematic. A 2-fold change in the concentration of ^18^O_2_ 5-InsP_7_ decreased skewing by 2%. Comparison of the 5-InsP_7_ results based on unit mass resolution with wide mass resolution results in yeast samples demonstrate a similar trend in ^18^O labeling overtime (Figures S14 and S15), indicating the skewing does not impact the trend used to assess the dynamic turnover of phosphorylated InsPs and PP-InsPs in this study. In summary, ^18^O labeling of the inositol pyrophosphates InsP_7_ and InsP_8_ is moderately overestimated in the wide mass resolution approach. This error must be accepted as a trade-off for attaining the enhanced sensitivity that is necessary for analyzing ^18^O transfer to very low-abundance PP-InsPs, such as InsP_8_. Consequently, the following results were obtained using wide mass resolution CE-QQQ, except for 5-InsP_7_ in yeast (Figures 5B and S17B).

Next, the turnover of the inositolpyrophosphates, particularly 5-InsP_7_ and 1,5-InsP_8_, was studied in cells under constant nutrient availability. When the cells were transferred from unlabeled culture medium into medium of the same composition but made from 50% ^18^O-water, 12% of 5-InsP_7_ and 44% of 1,5-InsP_8_ acquired one or two ^18^O labels already within 1 min (Figure 5A,B, for representative examples of extracted ion electropherograms see Figure S16). For 1,5-InsP_8_, this led to 78% labeling of the pool within 15 min and remained almost constant thereafter. Labeled 5-InsP_7_ attained 46% within the first 15 min, which then slowly increased for the rest of the 60 min incubation period (50% of labeled 5-InsP_7_). This suggests the existence of at least two pools of 5-InsP_7_, one resting static and the other one turning over very vigorously. 100% ^18^O-water labeling results confirmed our hypothesis by showing complete labeling of the 1,5-InsP_8_ pool within 30 min while 5-InsP_7_ maintained a plateau between 60 and 70% (Figure S17). In contrast to the inositol pyrophosphates, InsP_6_ was labeled only very slowly, showing 12% ^18^O labeling after 60 min (Figure 5C). This suggests that the InsP_6_ pool is relatively static regarding its synthesis from InsP_5_, whereas the inositol pyrophosphates undergo a highly dynamic, permanent turnover of their phosphate groups. Since both PP-InsPs are synthesized from InsP_6_, turnover of their phosphate groups can be attributed almost exclusively to the exchange of the β-phosphates of their diphosphate groups. Remarkably, this high turnover occurs even under constant nutrient availability. It hence appears to be a constitutive feature of inositol pyrophosphate metabolism.

The analysis of lower phosphorylated InsPs using the same experimental approach revealed further interesting behavior. InsP_3_, which is generated from PtdIns­(4,5)­P_2_ through phospholipase C (Figure 1), incorporated ^18^O into ca. 50% of the pool within 1 min (Figure 5G,H). The InsP_3_ pool could be dissected into two different species with similar dynamics. To assign the InsP_3_ isomers, a homemade [^13^C_6_] InsP_3_ reference solution was generated by incubating [^13^C_6_] InsP_6_ (prepared with ultrapure water) at 100 °C for 5 h.^50^ The assignment of each generated [^13^C_6_] InsP_3_ isomer was achieved by a previously described method.^17^ One of the ^18^O-InsP_3_ species that we observed has the same electrophoretic migration time as Ins­(1,4,5)­P_3_, Ins­(1,3,4)­P_3_, and Ins­(1,4,6)­P_3_ (Figure S18), which cannot be differentiated with our method. Considering published data,^18,51,52^ we infer that its identity is most likely Ins­(1,4,5)­P_3_, the direct product of PtdIns­(4,5)­P_2_ hydrolysis. The other labeled InsP_3_ species does not comigrate with any reference InsP_3_ isomer that is available in our laboratory. Since the only InsP_3_ reference for which this applies is Ins­(2,4,6)­P_3_, we tentatively assign this species as Ins­(2,4,6)­P_3_. Interestingly, the incorporation of up to five ^18^O labels into InsP_3_ was detected by CE-qTOF, suggesting that several phosphate groups undergo exchange. This is in stark contrast to what we observed for, e.g., InsP_6_ or InsP_7_. For Ins­(1,4,5)­P_3_, this highly dynamic turnover may occur while it still forms the headgroup of PtdIns­(4,5)­P_2_, but Ins­(2,4,6)­P_3_ does not directly derive from this lipid^51^ and might undergo lipid-independent turnover (Figures 5I,J, and S19).

InsP_4_ showed comparably limited turnover (Figure 5E,F) and could be dissected into two different InsP_4_ species. One of them, assigned as Ins­(1,3,4,5)­P_4_ (Figure S20), was static after 1 min and incorporated ^18^O to ca. 20%. The other one, which currently cannot be unambiguously assigned, showed very sluggish but continuous ^18^O incorporation. The different pools of InsP_4_ very likely represent positional isomers with regard to the phosphate groups and not diastereomeric inositol core structures, such as *scyllo* inositol.

Combining the results with observations of the different dynamic phosphate exchanges among various InsPs in yeast, we propose a three-cycle model in which phosphate cycling occurs on discrete compounds along the inositol phosphate metabolic pathway (Figure 5K). For example, in the cycle of InsP_6_ and inositol pyrophosphates (5-InsP_7_; 1,5-InsP_8_), highly dynamic phosphate conversion from InsP_6_ to 5-InsP_7_ was observed and 5-InsP_7_ further interconverts with 1,5-InsP_8_. In this cycle, only the β-phosphates are dynamic.

Inositol pyrophosphates are key regulators of P_i_ homeostasis. Their levels decrease under P_i_ starvation and inhibiting their synthesis induces a constitutive P_i_ starvation response.^53–56^ The synthesis of these inositol pyrophosphates is regulated by intracellular P_i_ and by ATP levels, which is strongly correlated with P_i_ availability. We hence used ^18^O labeling to investigate differences in inositol pyrophosphate turnover during P_i_ starvation.^53^ As shown in Figure 6A, both 5-InsP_7_ and 1-InsP_7_ exhibited lower ^18^O incorporation under P_i_-starved conditions as compared to those under P_i_-rich conditions. This difference appeared as early as 1 min and became more pronounced over time. By contrast, InsP_6_, which remains relatively stable during P_i_ starvation, showed little difference in ^18^O labeling. These results illustrate the rapid and selective impact of P_i_ starvation on the turnover of higher inositol pyrophosphates on a minute time scale.

**Figure 6:**
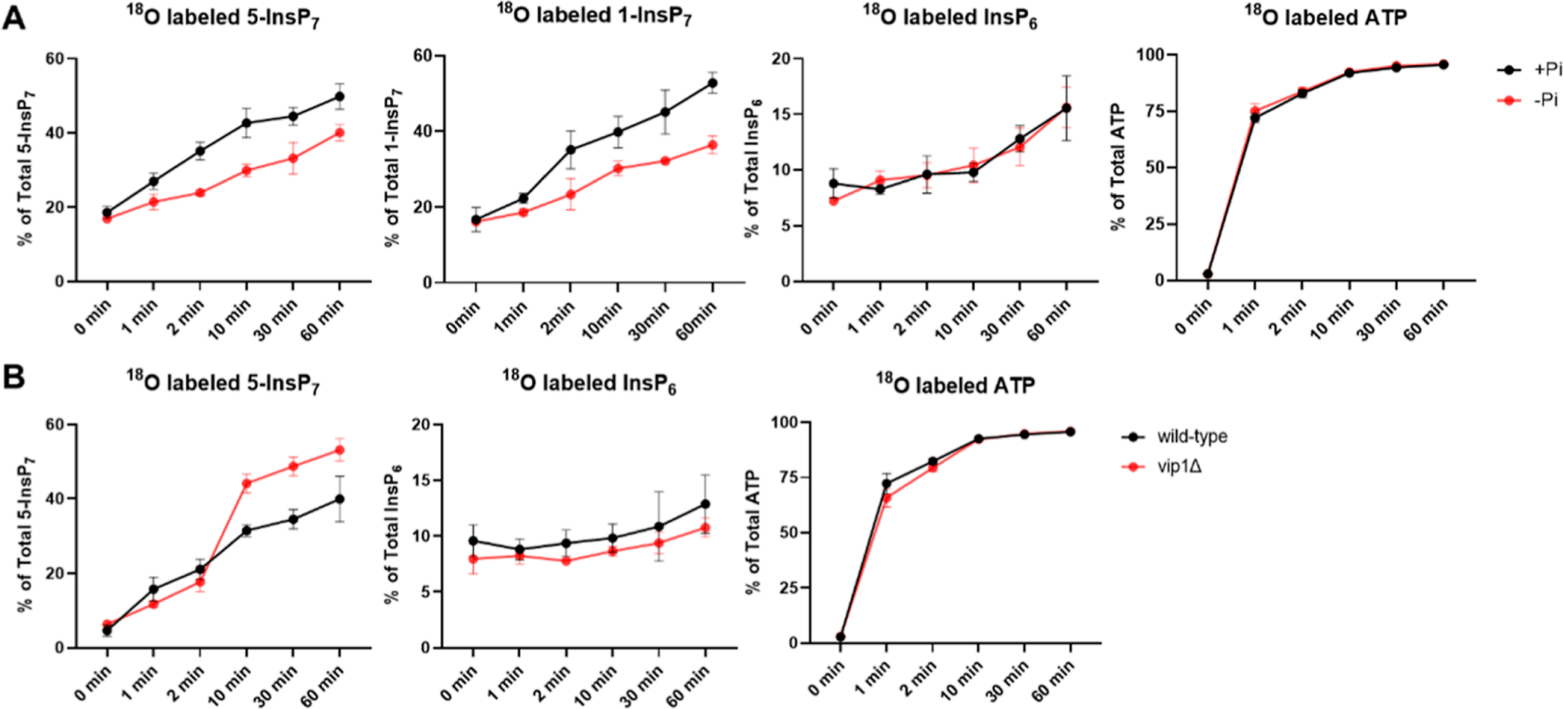
Kinetics of ^18^O entry into soluble InsPs and ATP of yeast under different Pi conditions and in vip1Δ mutant. Yeast cells grown in P_i_-rich medium were transferred to P_i_-rich or P_i_-free media containing 50% ^18^O-labeled water, and aliquots were analyzed at the indicated time points following the transfer. Cells were extracted with perchloric acid and TiO_2_ and analyzed by CE-QQQ. The means of 3 samples with standard deviation is shown. (A) ^18^O labeled 5-InsP_7_ (sum of ^18^O_1_ and ^18^O_2_), 1-InsP_7_ (sum of ^18^O_1_ and ^18^O_2_), InsP_6_ (sum of ^18^O_1_ and ^18^O_2_), and ATP (sum of ^18^O_1_ to ^18^O_7_) in the wild-type yeast under different Pi conditions. ATP was measured by CE-QQQ with unit mass resolution, whereas the others were measured by CE-QQQ with wide mass resolution. (B) ^18^O labeled 5-InsP_7_ (sum of ^18^O_1_ and ^18^O_2_), InsP_6_ (sum of ^18^O_1_ and ^18^O_2_), and ATP (sum of ^18^O_1_ to ^18^O_7_) in the wild-type and vip1Δ mutant under P_i_-rich condition. ATP and 5-InsP_7_ were measured by CE-QQQ with unit mass resolution, whereas the others were measured by CE-QQQ with wide mass resolution.

We also compared the ^18^O labeling in a vip1Δ mutant that fails to synthesize InsP_8_ and 1-InsP_7_ but accumulates more than 10-fold higher 5-InsP_7_ levels.^53^ Under P_i_-rich conditions, ^18^O labeled 5-InsP_7_ was accumulated to a greater extent in the vip1Δ mutant compared to the wild type, particularly after 10 min (Figure 6B), whereas the ratio of ^18^O labeled InsP_6_ to unlabeled InsP_6_ remained similar. ^18^O incorporation into the ATP pool remained the same in both strains (wild type and vip1Δ) (Figure 5).

### 2.3 Kinetic Compartmentalization of the InsP Pathway in Human Cells

Next, we tested the suitability of ^18^O-water to label inositol phosphates in HCT-116 cells. These experiments employ larger volumes of media and were hence performed with 50% ^18^O-water only to reduce the cost. Cells were pulse-labeled by transferring them into medium made with 50% ^18^O-water but of otherwise identical composition. At different time-points of pulse-labeling, aliquots were extracted with perchloric acid and analyzed by CE-MS (Figure 7).

**Figure 7:**
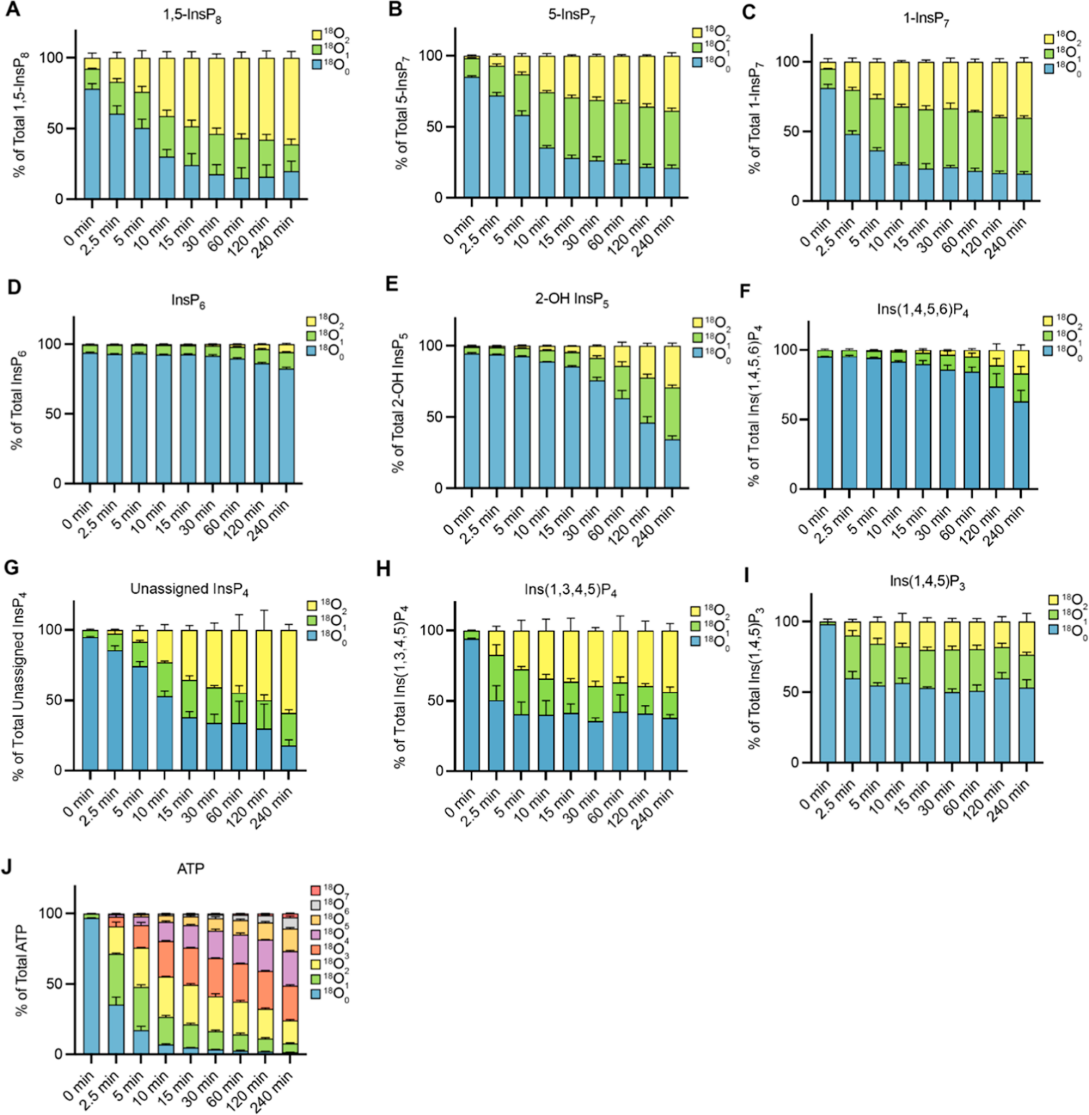
Kinetics of ^18^O entry into ATP and cytosolic InsPs from human cells. Kinetics of ^18^O entry into 1,5-InsP_8_ (A), 5-InsP_7_ (B), 1-InsP_7_ (C), InsP_6_ (D), 2-OH InsP_5_ (E), Ins­(1,4,5,6)­P_4_ (F), unassigned InsP_4_ (G), Ins­(1,3,4,5)­P_4_ (H), Ins­(1,4,5)­P_3_: Ins­(1,4,5)­P_3_ (I), and ATP (J) were monitored in mammalian cells. HCT116 cells were grown in DMEM medium, detached, and collected by centrifugation. The cell pellet was resuspended in DMEM medium prepared with 50% of ^18^O-labeled water. After the indicated periods of further incubation at 37 °C, aliquots of the cells were extracted with perchloric acid. CE-MS analyses of the extracts were performed and rare analytes (PP-InsPs) were measured in wide mass resolution. The means of three replicates are shown with standard deviations.

^18^O rapidly entered ATP and the inositol pyrophosphates (5-InsP_7_; 1-InsP_7_; 1,5-InsP_8_; data collected in wide mass resolution), reaching equilibrium after 10–20 min. Between 20 and 25% of these PP-InsPs remained unlabeled. InsP_6_ labeling proceeded slowly also in mammalian cells, reaching only 1/6th of the pool after 4 h. This incorporation probably reflects new synthesis of InsP_6_ through cell growth because the generation time of the cells under these steady media conditions is around 24 h. During the 4 h of labeling time, biomass would thus increase by roughly 1/6th, accounting for the increase in labeled InsP_6_, which is of the same order. Interestingly, 2-OH InsP_5_, which is of similar abundance as InsP_6_ in some mammalian cells,^45^ showed significantly faster labeling than InsP_6_, suggesting that the flux from InsP_5_ to InsP_6_ by the inositol-pentakisphosphate 2-kinase (IPPK) is sluggish. The appearance of only two labels suggests that turnover takes place at a specific position and not at many different positions, as this would lead to higher numbers of ^18^O incorporations.

Each InsP_4_ isomer showed different ^18^O incorporation patterns. Ins­(1,4,5,6)­P_4_ (assignment shown in Figure S20), which is not observed in yeast, exhibited relatively slow incorporation of ^18^O, similar to InsP_6_, whereas 80% of a currently unassigned InsP_4_ was labeled to more than 80% with ^18^O over 4 h. This unassigned InsP_4_ is different from the unassigned InsP_4_ in yeast. Similarly as in yeast, Ins­(1,3,4,5)­P_4_ incorporated ^18^O very rapidly, reaching a plateau within 2.5 min that remained constant for the rest of the 4 h incubation period.

We could also resolve three peaks of InsP_3_. By comparison with ^13^C-labeled internal references (Supplementary Figure S18), we assign the first one as Ins­(1,2,3)­P_3_. The second peak remains ambiguous (Ins­(1,2,6)­P_3_ and/or its enantiomer Ins­(2,3,4)­P_3_). Both signals are apparently not significantly generated through kinases, which would lead to incorporation of the ^18^O label. They might, however, originate from dephosphorylation of InsP_6_ into these metabolites (see the Discussion). Since generation of Ins­(1,2,6)­P_3_ through dephosphorylation by MINPP1 was recently described using ^13^C NMR labeling,^15^ the second peak thus likely represents Ins­(1,2,6)­P_3_.

The third peak was assigned as Ins­(1,4,5)­P_3_ as the most likely species. It incorporated ^18^O very rapidly and similarly as Ins­(1,3,4,5)­P_4_ attained a plateau within 2.5 min that remained constant for the rest of the 4 h incubation. As in yeast, up to five ^18^O labels of Ins­(1,4,5)­P_3_ were detectable by CE-qTOF within 15 min, suggesting turnover of multiple phosphate groups (Figure S21). Unfortunately, we could not record these multiple ^18^O labels accurately beyond 15 min because of matrix effects in these sample sets. Labeling did not reach the entire pool of Ins­(1,4,5)­P_3_. Thus, similarly as argued above for yeast, there might be subpools of this compound, some of which remain quite static and others that turn over rapidly and are responsible for the initially rapid yet limited integration of ^18^O into the total pool of Ins­(1,4,5)­P_3_.

Together, these data suggest massively different dynamics of phosphate exchange on InsPs also in mammalian cells: highly dynamic phosphate cycling of the P-anhydrides in the inositol pyrophosphate pools (5-InsP_7_; 1-InsP_7_; 1,5-InsP_8_) and of phosphate esters in InsP_3_ - likely resulting from the lipid-dependent turnover and cleavage by PLC—is separated by a much more inert pool of InsP_6_. 2OH-InsP_5_ shows an intermediate behavior, and for InsP_4_ turnover can be very rapid or sluggish, depending on the isomer. Thus, the inositol pyrophosphate and InsP_3_ pools may cycle independently from each other, be connected through slower biosynthetic reactions, or be separated by compartmentalization.

### 2.4 ^18^O-Water Pulse Labeling Reveals a Sluggish PP-InsPs Metabolism in Amoeba

Finally, we performed similar labeling and extraction experiments with 50% ^18^O-water with the slime mold Dictyostelium discoideum (Figure 8). This organism has played an important role in the discovery of PP-InsPs as its concentrations are comparably high, reaching submillimolar levels.^57^ It produces a range of PP-InsP isomers different from those found, for example, in yeast and mammals.^14,58^ In D. discoideum, around 75% of total ATP had incorporated ^18^O within 10 min of incubation (Figure S22). These proportions changed only a little in the subsequent 20 min, suggesting the existence of two pools of ATP that turn over at different velocities.

**Figure 8:**
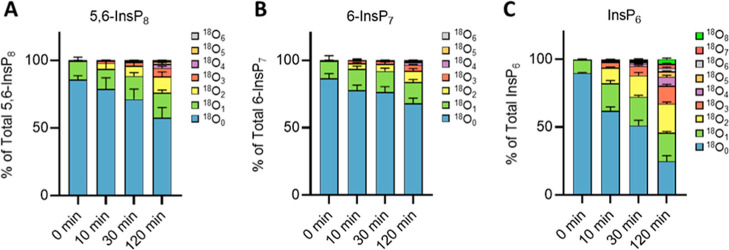
Kinetics of ^18^O entry into soluble InsPs from D. discoideum. Kinetics of ^18^O entry into 5,6-InsP_8_ (A,) 6-InsP_7_ (B), and InsP_6_ (C) monitored in Dictyostelium. Cells were precultured in SIH medium and transferred to SIH media made of 50% of ^18^O-labeled water with P_i_. After further incubation for the indicated periods of time, samples were harvested and extracted. Extracts were measured by CE-QQQ with wide mass resolution. The means of four replicates with standard deviations are shown.

Labeling of InsP_7_ and InsP_8_ was much slower than in yeast and human cells: only 32% InsP_7_ and 44% InsP_8_ incorporated labels throughout the 2 h of incubation time. Considering the approximately 8 h generation time of Dictyostelium, the rate of incorporation into InsP_7_ may reflect new biosynthesis due to growth of the culture. The labeling of InsP_8_ goes beyond that but is far from the vigorous turnover observed in yeast and mammalian cells. InsP_6_ labeling, on the other hand, proceeded much faster than that of the inositol pyrophosphates, and it was also more rapid and extensive than the labeling of InsP_6_ in yeast and human cells. Up to eight ^18^O labels were detected throughout the experiment, necessitating rapid phosphate group exchange at multiple positions in InsP_6_. Thus, not only are the dynamics of InsPs metabolism in Dictyostelium significantly different than in budding yeast or mammalian cells but also the number of phosphate groups that undergo exchange. Unfortunately, the sensitivity needed to obtain a comprehensive analysis of the labeling of InsP_5_, InsP_4_, and InsP_3_ was affected by the abundant and broad ethylenediaminetetraacetic acid (EDTA) signal present from the extraction buffer. For this reason, we do not report lower InsPs from these samples.

## 3 Discussion

The metabolism of InsPs and PP-InsPs, which are signaling molecules of low abundance, has mainly been studied using radioactive labeling of inositol or of phosphate.^13,19^ For inositol poly- and pyrophosphates, the labeling approach with ^18^O-water that we present here offers several important advantages, particularly for analyzing turnover of the phosphate groups.^34,37,59^ ^18^O is a stable, nonradioactive isotope, such that media and samples produced with it can be stored for extended periods of time. ^18^O-water can be obtained in a 99.5% pure form, enabling labeling to a very high specific isotope content. This facilitates detection of inositol phosphates by mass spectrometry, some of which exist in submicromolar concentrations in cells.^50,53,60,61^ ^18^O-water labeling can be performed in any medium and under any growth condition. This is a distinctive advantage over labeling with radioactive inositol. Cellular membranes are also permeable to water, which allows very rapid labeling (seconds, as compared to hours for inositol labeling) to high specific isotope content and apparent equilibrium. In combination with CE-MS detection, this facilitated the analysis of an unexpectedly rapid turnover of phosphate groups around the inositol ring with high specificity. Turnover can be monitored simultaneously for a variety of InsPs in the same experiment.

The possibility of monitoring the kinetics of phosphate group turnover on InsPs with improved temporal resolution, sensitivity, throughput, and cost renders it possible to analyze flux through the metabolic pathways interconnecting inositol phosphates. Our method can detect inositol phosphates with high accuracy if applied in the unit-resolution or wide-resolution mode for singly charged ions. For doubly charged anions, wide resolution produces a skewing from the true value that is on the order of 10% and unit mass resolution is preferable. However, under these conditions, the sensitivity of the wide mass resolution method is higher, which enables monitoring of metabolites and isotopologues of low abundance. The method also allows labeling of inositol phosphate lipids. We currently exploit this to derive metabolic network models for inositol polyphosphate metabolism and to explore the dynamics of inositol lipids, aspects that will be described in separate studies. One such network model for the inositol pyrophosphates has now become available.^62^

Our analysis relies on the very high turnover of ATP inside cells, which renews and labels the entire ATP pool at the time scale of seconds to a few minutes, depending on the cell type.^42^ Label can then be transferred to inositol phosphates by the respective kinases, and it can be removed again by hydrolysis through phosphatases. The efficiency of this ^18^O-water labeling approach is illustrated by the rapid labeling kinetics that we could uncover for some compounds in yeast, such as the PP-InsPs and some InsP_3_ species (Figure 5K). Other pools of inositol phosphates, such as InsP_6_ and InsP_4_, incorporated ^18^O very slowly, suggesting at first sight a very low turnover. It is worthwhile to consider the enzymatic aspect of this process. Kinases transfer phosphate from ATP through a variety of mechanisms.^63^ A common feature is that the γ-phosphate of ATP undergoes an attack, for example, by an OH group of the substrate to be phosphorylated. When ADP leaves, the oxygen constituting the link to the phosphorylated substrate stems from the unlabeled substrate, not from the ^18^O labeled γ-phosphate group. If the substrate is dephosphorylated again, labeled water attacks the phosphate group and the substrate leaves, keeping its unlabeled oxygen.^64^ Label is thus introduced by the kinase reaction and withdrawn by the opposing phosphatase reaction. We may then expect that the substrate is ^18^O labeled only as long as it maintains the labeled phosphate group. Therefore, InsP_6_ should not be considered a truly static pool. The rapid labeling of 5-InsP_7_ through InsP_6_ kinase requires cycling of the β-phosphate on position 5 of the inositol ring and engages InsP_6_ as a substrate. Thus, there should be rapid cycling between InsP_6_ and 5-InsP_7_ by phosphorylation and dephosphorylation, but it does not leave a corresponding ^18^O mark on InsP_6_. Along the same lines, we expect the labeling of 2OH-InsP_5_ to stem from the phosphorylation of InsP_4_ through inositol polyphosphate multikinase. That this accumulating label is propagated only very inefficiently from 2OH-InsP_5_ into InsP_6_ argues for a sluggish exchange between InsP_5_ and InsP_6_ under the conditions we investigated. For similar reasons, we expect the interconversion of InsP_3_ and InsP_4_ to be slow (Figure 5K).

These very pronounced differences in turnover suggest that phosphate cycling can occur on discrete compounds along the inositol phosphate metabolic pathway. This can separate the rapidly interconverting InsP_7_ and InsP_8_ from sluggish interconversions between InsP_3_ and InsP_4_ and between InsP_5_ and InsP_6_ (Figure 5K). It suggests that even though InsP_4_ through InsP_8_ derive from InsP_3_ and enzymes can interconvert all these compounds (Figure 1),^18,52,65^ they do not operate as a simple linear pathway. Instead, InsPs appear to be organized into a series of separated phosphate cycles. In this way, each cycle is free to mediate independent signaling processes. The metabolic reactions connecting the cycles may then serve mainly to replenish or diminish substrate levels in the cycle, for example, when growth or changing nutrient conditions may necessitate an adaptation of the signaling properties. Such a scenario arises, for example, upon phosphate replenishment after starvation, when the InsP_7_ and InsP_8_ pools become significantly expanded.^6,53–55^

Turnover of PP-InsPs and InsP_3_ is very vigorous, even under steady growth conditions, i.e., under constant nutrient supply and growth. Such strong turnover implies permanent competing synthesis and degradation of the compounds. The simultaneous activity of two antagonizing reactions is often considered as “wasteful cycling”. It may, however, be worth the price because it comes with significant benefits and additional potential for information transmission. Substrate cycling can sharpen the responses of a signaling system and increase its sensitivity. It can amplify signals,^66,67^ allow to filter or process information from noise in external and internal parameters, and create bistable switches.^68,69^ This makes, for example, the rapid cycling of 1,5-InsP_8_ interesting and potentially relevant because the yeast PHO pathway is controlled by 1,5-InsP_8_,^53^ exploits expression noise, and behaves like a bistable switch.^70–73^

Some inositol phosphates, such as 5-InsP_7_ in yeast or InsP_3_ in human cells, showed biphasic labeling kinetics, where a rapid initial turnover was followed by a long phase in which no or only negligible further ^18^O was incorporated. Since the cells had been growing under steady conditions, it is unlikely that this reflects a sudden change in the metabolism of these compounds. A more plausible explanation is sequestration of the compound into pools of greater or lesser accessibility to metabolizing enzymes. Such sequestration can be realized by stable binding of InsPs to proteins^74^ or other molecules and/or by transfer into distinct subcellular compartments. For InsPs, several examples show that they can integrate into proteins in an apparently very stable fashion such that the interaction persists even through purification and crystallization of the proteins. Examples include the capsid and Gag proteins of retroviruses,^75–77^ the RNA editing enzyme ADAR2, or casein kinase 2.^78,79^ Moreover, InsPs might be sequestered into biomolecular condensate type structures such as nucleoli.^80^ Such mechanisms may also localize InsPs to cytosolic areas with greater or lesser availability of labeled ATP because the distribution of ATP in the cytosol is inhomogeneous.^81–83^

Another, equally interesting possibility is that subpools of the same compound show different turnover rates because they reflect heterogeneities in the cell population. Cell populations, even those of simple unicellular organisms, are heterogeneous. The cells are in different metabolic, signaling, and cell cycle states, which can sometimes be quite stably maintained to ensure diversity in the population.^84^ Then, different cell states may give rise to differences in InsP turnover, which we revealed here. Some InsPs undergo indeed significant changes during the cell cycle.^61,85^ Investigating such heterogeneities systematically becomes accessible with our approach since it is nonradioactive and sensitive, thus allowing to sort cell populations into specific subpools, for example, by fluorescence-activated cell sorting, to dissect the diversity in their InsPs metabolism.

In addition to analyzing the turnover of individual InsPs using the ^18^O labeling method, the approach can also be applied to understand how InsPs dynamics change under different physiological conditions, such as those shown for P_i_-rich vs P_i_-depleted conditions (Figure 6A) or in KO cell lines, such as vip1Δ (Figure 6B). For example, we observe an increased turnover of inositol pyrophosphates under phosphate-rich conditions as compared with phosphate-depleted conditions, while InsP_6_ and ATP turnover remain unchanged. Rapid pulse labeling by ^18^O-water followed by CE-MS allows to generate kinetic data of significantly better time and isomer resolution than the traditional radioactive approaches.^13^ Combining this approach with controlled changes in growth conditions or the acute stimulation or inhibition of signaling pathways should allow us to obtain novel insights into the regulation and signaling properties of inositol phosphate pools. It will help us to create metabolic models of inositol phosphate-based signaling pathways and to better understand effects exerted by kinase inhibitors on cellular inositol phosphate fluxes, an area of growing interest in, for example, metabolic diseases.^86^ Indeed, the data sets obtained herein have already served as the basis to create mathematical models of the inositol pyrophosphate network.^62^

In striking contrast to the situation in yeast and human cells, we observed a sluggish PP-InsP turnover but unexpectedly high turnover of InsP_6_ occurring on many of its different phosphate groups in the amoeba D. discoideum. Its lethargic PP-InsP turnover suggests that the amoeba employs these metabolites differently than yeast or mammalian cells. This correlates with the diversification of the InsP-kinase families that are used in different eukaryotic clades to drive InsPs metabolism and signaling^87^ and with the complexity of the enzymology of PP-InsPs metabolism in amoeba, which is far greater than in yeast or mammalian cells.^58^ The strikingly different behavior of Dictyostelium calls for a detailed analysis of InsP fluxes, which may uncover new strategies for InsP signaling. The methods presented here open the door to such investigations.

## 4 Material and Methods

### 4.1 Cell Strains and Culture Media

#### 4.1.1 Yeast

The Saccharomyces cerevisiae strain BY4741 (*MATa his3*Δ*1 leu2*Δ*0 met15*Δ*0 ura3*Δ*0*) was used in this study. The vtc4Δ and vip1Δ mutant strains were used as in the previous studies, ref 88 and ref 53, respectively. Yeast cells were shaken (150 rpm, 30 °C, unless stated otherwise) in SC medium: 6.7 g/L yeast nitrogen base (Formedium, USA) and 2% of glucose.

Medium for ^18^O labeling was prepared as a 2× concentrated SC stock made with normal water in advance, which was then diluted by an equal volume of 99% ^18^O-water (Medical Isotopes Inc.) to make SC with 50% ^18^O-water. The P_i_-free medium was prepared using yeast nitrogen base without phosphate (Formedium, USA).

#### 4.1.2 Mammalian Cells

HCT116^UCL^ cells were used in this study. Cells were grown for 6 passages in DMEM-HAM’s F12 (Gibco) supplemented with 10% (v/v) fetal calf serum, 50 U/ml penicillin, 50 mg/mL streptomycin, 5 μg/mL insulin. Cells were grown at 37 °C in 5% CO_2_ with 98% humidity.

For ^18^O labeling, a 2× concentrated medium was prepared using DMEM powder (Sigma) supplemented with 7.4 g/L sodium bicarbonate. This stock was diluted by an equal volume of ^18^O-labeled water to yield the medium with 50% ^18^O-water that was used for the experiments.

#### 4.1.3 Dictyostelium

D. discoideum strain AX2 obtained from dictyBase (http://dictybase.org) was used in this study. Amoeba were grown at 22 °C with gentle shaking (100 rpm) in synthetic SIH medium (Formedium, #SIH0101). Medium for ^18^O labeling was prepared as a 2× concentrated SIH stock made with ddH_2_O, which was then diluted by an equal volume of 99% ^18^O-water (Medical Isotopes Inc.) to make SIH with 50% ^18^O-water.

### 4.2 Extraction of InsPs and ATP

#### 4.2.1 Yeast

Yeast cells were grown at 20 °C overnight in 50 mL of SC medium to reach logarithmic phase (4.3 × 10^7^ cells/ml) in the morning. 4 ml samples were harvested by centrifugation (3200*g*, 3 min, 20 °C) and resuspended in the same volume of SC prepared with 50% of ^18^O-labeled water. Cells were further incubated under the same conditions.

3 ml of yeast culture (4.3 × 10^7^ cells/ml) was mixed with 300 μL of 11 M perchloric acid to a final concentration of 1 M. Samples were snap-frozen in liquid nitrogen and then centrifuged at 20,000*g* for 3 min at 4 °C. The soluble supernatant was transferred to a new tube, and each sample was supplemented with 6 mg of titanium dioxide (TiO_2_) beads (GL Sciences, Japan), which had been prerinsed through two rounds of washing with 1 mL of H_2_O and 1 M perchloric acid, respectively. The mixture was gently rotated for 15 min at 4 °C and centrifuged at 20,000*g* for 3 min at 4 °C. The TiO_2_ beads were washed twice using 500 μL of 1 M perchloric acid. InsPs and ATP were eluted by incubating the beads with 300 μL of 3% (v/v) NH_4_OH for 5 min at room temperature under gentle shaking. After centrifugation at 20,000*g* for 3 min, the eluents were transferred to a new tube. Any remaining TiO_2_ beads were removed by centrifugation at 20,000*g* for 3 min. The resulting supernatant was completely dried in a SpeedVac (Labogene, Denmark) at 42 °C. Samples were kept at −20 °C until analysis.

For experiments under P_i_-rich and P_i_-starvation conditions, yeast cells were first grown in a P_i_-rich medium at 20 °C overnight. Upon reaching the logarithmic phase, the culture was split into two and washed twice with either a P_i_-rich or P_i_-free medium prepared with normal water. Cells were then resuspended in an ^18^O-labeled P_i_-rich or ^18^O-labeled P_i_-free medium. Samples were harvested at different time points using the same procedure and InsPs were extracted as described above.

#### 4.2.2 Human Cells

HCT116^UCL^ cells were seeded in 10 cm^2^ Petri dishes and grown to 80% confluence (6.5 × 10^6^ cells) at 37 °C as described above. Cells were detached from the Petri dish by adding 5 mL of 0.25% Trypsin (Thermo Fisher Scientific, USA) and harvested by centrifugation at 3200*g* for 5 min. The cell pellet was resuspended in 1 mL of DMEM medium (Thermo Fisher Scientific, USA) prepared with 50% of ^18^O-labeled water and further incubated at 37 °C.

At different time points, 1 mL of samples were mixed with 100 μL of 11 M perchloric acid. After snap-freezing in liquid nitrogen, samples were centrifuged at 16,000*g* in a tabletop centrifuge. The soluble supernatant was transferred into a new tube and mixed with prewashed TiO_2_ beads (5 mg of beads per sample). The extraction was performed in the same way as for yeast.

#### 4.2.3 D. discoideum

D. discoideum cells were seeded at 2–5 × 10^5^ cells/ml and grown for 24–48 h in 50 mL of SIH medium to reach a cell density of 1–3 × 10^6^. Amoeba (8 × 10^6^ per experimental point) were transferred into a 15 mL falcon tube, harvested by centrifugation (800*g*, 5 min, 22 °C), and resuspended in 2 mL of SIH medium prepared with 50% of ^18^O-water. Cells were further incubated under the same conditions. At specific time points, the falcon tube was spun (1000*g*, 2 min, 4 °C) and the cell pellet snap-frozen in liquid nitrogen. At the end of the experiment, each cell pellet was resuspended in 1 M perchloric acid with 5 mM EDTA and InsPs subjected to TiO_2_ purification as previously described.^47,48^

### 4.3 Potential ^16/18^Oxygen Exchange

#### 4.3.1 ATP in the Presence of ^18^O-Water or Inositol Hexakisphosphate Kinase IP6K1

After incubating 1 mM of ATP with ^16^O-water, 50% of ^18^O-water (>98 atom % ^18^O), IP6K1 reaction buffer (50 mM NaCl, 20 mM HEPES, 6 mm MgCl_2_, 1 mM DTT, pH 6.8), and purified IP6K1 (0.0375 μg/μL)^89^ at 37 °C for 4 h, samples were diluted 2-fold and measured by CE-QQQ with unit mass resolution.

#### 4.3.2 InsPs and PP-InsPs in the Presence of ^18^O-Water

After incubating 4 μM of Ins­(1,4,5)­P_3_, Ins­(1,3,4,5)­P_4_, 2-OH InsP_5_, InsP_6_, 5-InsP_7_, and 1,5-InsP_8_ with 50% of ^18^O-water (>98 atom % ^18^O) at 37 °C for 4 h, samples were measured by CE-QQQ with unit mass resolution.

#### 4.3.3 InsPs and PP-InsPs in the Presence of Inositol Hexakisphosphate Kinase IP6K1

After incubating 100 μM of InsP_6_ or 5-InsP_7_ with 50% ^18^O-water (>98 atom % ^18^O), IP6K1 reaction buffer (50 mM NaCl, 20 mM HEPES, 6 mm MgCl_2_, 1 mM DTT, pH 6.8), and purified IP6K1 (0.0375 μg/μL) at 37 °C for 4 h, samples were diluted 2-fold and measured by CE-QQQ with unit mass resolution.

#### 4.3.4 Extraction of InsPs and PP-InsPs in the Presence of ^18^O-Water

4 μM of InsP_6_, 5-InsP_7_, 1-InsP_7_, and 1,5-InsP_8_ were added to 800 μL of 1 M perchloric acid containing 50% (v/v) of ^18^O-water and InsPs subjected to TiO_2_ purification as described above. Samples were then measured by CE-QQQ with a unit mass resolution.

#### 4.3.5 IP6K1 In Vitro Assay

100 μM InsP_6_ was incubated with 1 mM ATP or ^18^O_2_ ATP, IP6K1 reaction buffer (50 mM NaCl, 20 mM HEPES, 6 mm MgCl_2_, 1 mM DTT, pH 6.8), and purified IP6K1 (0.0375 μg/μL) in the presence of 50% (v/v) of ^18^O-water or 100% of ^16^O-water at 37 °C for 4 h. Then, samples were placed on ice, and the enzymatic activity was quenched by adding 10 μM (working concentration) EDTA. After 2-fold dilution, samples were measured by CE-QQQ with unit mass resolution.

### 4.4 CE-ESI-MS Analysis of InsPs, PP-InsPs, and ATP

#### 4.4.1 CE-qTOF

An Agilent 7100 capillary electrophoresis system coupled to a qTOF (Agilent 6520) equipped with a commercial CE-MS adapter and sprayer kit (from Agilent) was used. The sheath liquid (water: isopropanol = 1:1, v/v) spiked with mass references (TFA anion, [M – H]^−^, 112.9855; HP-0921, [M – H + CH_3_COOH]^−^, 980.0163) was introduced with a constant flow of 6 μL/min. A bare fused silica capillary with a length of 100 cm (50 μm internal diameter and 365 μm outer diameter) was used for CE separation. The background electrolyte (35 mM ammonium acetate titrated with ammonium hydroxide to pH 9.75) was employed for all of the experiments. Before the run, the capillary was flushed with background electrolytes for 400 s. 30 or 40 nL of sample was injected by applying pressure (100 mbar for 15 or 20 s). The qTOF was conducted in negative ionization mode. MS source and scan parameters shown in Table S1. Biological samples were analyzed by Acquisition mode MS1. For the ESI-MS fragmentations of ATP and 5-InsP_7_, Acquisition mode Auto MS2 was employed.

#### 4.4.2 CE-QQQ

We used an Agilent 7100 capillary electrophoresis system coupled to an Agilent 6495C Triple Quadrupole system, adopting an Agilent CE-MS interface. The sheath liquid (water/isopropanol = 1:1, v/v) was introduced with a constant flow of 10 μL/min. A bare fused silica capillary with a length of 100 cm (50 μm internal diameter and 365 μm outer diameter) was used for CE separation. The background electrolytes (35 mM ammonium acetate titrated with ammonium hydroxide to pH 9.75) was employed for all experiments. Before running the samples, the capillary was flushed with background electrolytes for 400 s. 30 nL of sample was injected by applying pressure (100 mbar for 15 s). Negative ionization mode was employed. MS source parameters are shown in Table S2. For yeast and HCT116 cell samples, MRM transition settings are shown in Tables S3, S4, and S5. For amoeba samples, MRM transition settings are shown in Tables S3, S6, S7, and S8. All samples are measured by CE-QQQ unless specifically stated otherwise.

## Supplementary Material



## Notes

Instrument and scan source parameters, MRM transitions settings of ATP and InsPs, oxygen migration (scrambling) on ATP and 5-InsP_7_ studies in the gas phase, extracted ion electropherograms of ATP and InsPs, spontaneous ^16/18^oxygen exchange studies, ^18^O labeling pattern of ATP and 5-InsP_7_ by CE-QQQ unit and wide resolution method, InsP_8_ and InsP_7_ labeling under 100% of ^18^O-labeled water, assignment of InsP_3_ and InsP_4_ isomers in yeast and HCT116 cells, qTOF analysis of InsP_3_ in yeast and HCT116 cells, kinetics of ^18^O labeling entry into ATP in D. discoideum, and additional references (PDF)
