# Supplementary material for: Pools of Independently Cycling Inositol Phosphates Revealed by Pulse Labeling with 18O‑Water
Source: J Am Chem Soc. 2025 May 15;147(21):17626–41. doi: 10.1021/jacs.4c16206 (PMC12123611; doi:10.1021/jacs.4c16206)
Supplement: Supplementary file 1 [file ja4c16206_si_001.pdf]

Pools of independently cycling inositol phosphates revealed by pulse labeling with ^{18}O-water

Geun-Don Kim[%1], Guizhen Liu[%2,3], Danye Qiu[2], Maria Giovanna De Leo[1], Navin Gopaldass[1], Jacques Hermes[3,4], Jens Timmer[3,4], Adolfo Saiardi[5], Andreas Mayer*[1], Henning Jacob Jessen*[2,3]

[1] *Département d'immunobiologie, Université de Lausanne, CH-1066 Epalinges, Switzerland*
[2] *Institute of Organic Chemistry, University of Freiburg, 79104 Freiburg, Germany*
[3] *CIBSS—Centre for Integrative Biological Signaling Studies, University of Freiburg, 79104 Freiburg, Germany*
[4] *Institute of Physics, University of Freiburg, 79104 Freiburg, Germany*
[5] *Medical Research Council, Laboratory for Molecular Cell Biology, University College London, WC1E 6BT London, United Kingdom*

[%]These authors contributed equally to this work.

*Corresponding authors: henning.jessen@oc.uni-freiburg.de; andreas.mayer@unil.ch

Supplementary Information

Supplementary Table S1. Instrument and scan source parameters of qTOF

**Instrument and scan source parameters**	
Gas Temperature	250 °C
Gas Flow	3 L/min
Nebulizer	10 psi
Vcap	3500 V
Fragmentor	100 V
Skimmer	65 V
OctopoleRFPeak	750 V
**Acquisition mode MS1**	
Scan *m/z* Range	80-1500
Scan Rate	1 spectra/sec
**Acquisition mode AutoMS2**	
Scan *m/z* Range	100-1000
Scan Rate	1 spectra/sec
Isolation Width MS/MS	Medium (˜4 amu)
Using Fixed Collision Energies	5 V, 10 V, 20 V, 30 V, 40V
Max Precursors per Cycle	2
Threshold	200 Abs
Threshold (Rel)	0.01
Sort Precursors	By abundance only
Charge State Preference	1, 2

Supplementary Table S2. Instrument and scan source parameters of QQQ

**Source Parameters**	
Gas Temperature	150 °C
Gas Flow	11 L/min
Nebulizer	8 psi
Sheath Gas Temperature	175 °C
Sheath Gas Flow	8 L/min
Capillary Voltage	-2000 V
Nozzle Voltage	2000 V
High Pressure RF (Ion Funnel Parameters)	70 V
Low Pressure RF (Ion Funnel Parameters)	40 V

Supplementary Table S3. MRM transitions setting of ATP for the measurement of ^{18}O labeled samples

Molecular name	Precursor Ion	Product Ion	Type of transition	Collision Energy (V)	Cell Accelerator Voltage
[$^{18}O_7$] ATP	520	420	$[M\text{-}H]^- \rightarrow [M\text{-}H\text{-}H_3PO_3{}^{18}O]^-$	25	1
[$^{18}O_7$] ATP	520	418	$[M\text{-}H]^- \rightarrow [M\text{-}H\text{-}H_3PO_2{}^{18}O_2]^-$	25	1
[$^{18}O_7$] ATP	520	416	$[M\text{-}H]^- \rightarrow [M\text{-}H\text{-}H_3PO^{18}O_3]^-$	25	1
[$^{18}O_7$] ATP	520	414	$[M\text{-}H]^- \rightarrow [M\text{-}H\text{-}H_3P^{18}O_4]^-$	25	1
[$^{18}O_6$] ATP	518	420	$[M\text{-}H]^- \rightarrow [M\text{-}H\text{-}H_3PO_4]^-$	25	1
[$^{18}O_6$] ATP	518	418	$[M\text{-}H]^- \rightarrow [M\text{-}H\text{-}H_3PO_3{}^{18}O]^-$	25	1
[$^{18}O_6$] ATP	518	416	$[M\text{-}H]^- \rightarrow [M\text{-}H\text{-}H_3PO_2{}^{18}O_2]^-$	25	1
[$^{18}O_6$] ATP	518	414	$[M\text{-}H]^- \rightarrow [M\text{-}H\text{-}H_3PO^{18}O_3]^-$	25	1
[$^{18}O_6$] ATP	518	412	$[M\text{-}H]^- \rightarrow [M\text{-}H\text{-}H_3P^{18}O_4]^-$	25	1
[$^{18}O_5$] ATP	516	416	$[M\text{-}H]^- \rightarrow [M\text{-}H\text{-}H_3PO_3{}^{18}O]^-$	25	1
[$^{18}O_5$] ATP	516	414	$[M\text{-}H]^- \rightarrow [M\text{-}H\text{-}H_3PO_2{}^{18}O_2]^-$	25	1
[$^{18}O_5$] ATP	516	412	$[M\text{-}H]^- \rightarrow [M\text{-}H\text{-}H_3PO^{18}O_3]^-$	25	1
[$^{18}O_5$] ATP	516	410	$[M\text{-}H]^- \rightarrow [M\text{-}H\text{-}H_3P^{18}O_4]^-$	25	1
[$^{18}O_4$] ATP	514	416	$[M\text{-}H]^- \rightarrow [M\text{-}H\text{-}H_3PO_4]^-$	25	1
[$^{18}O_4$] ATP	514	414	$[M\text{-}H]^- \rightarrow [M\text{-}H\text{-}H_3PO_3{}^{18}O]^-$	25	1
[$^{18}O_4$] ATP	514	412	$[M\text{-}H]^- \rightarrow [M\text{-}H\text{-}H_3PO_2{}^{18}O_2]^-$	25	1
[$^{18}O_4$] ATP	514	410	$[M\text{-}H]^- \rightarrow [M\text{-}H\text{-}H_3PO^{18}O_3]^-$	25	1
[$^{18}O_4$] ATP	514	408	$[M\text{-}H]^- \rightarrow [M\text{-}H\text{-}H_3P^{18}O_4]^-$	25	1
[$^{18}O_3$] ATP	512	414	$[M\text{-}H]^- \rightarrow [M\text{-}H\text{-}H_3PO_4]^-$	25	1
[$^{18}O_3$] ATP	512	412	$[M\text{-}H]^- \rightarrow [M\text{-}H\text{-}H_3PO_3{}^{18}O]^-$	25	1
[$^{18}O_3$] ATP	512	410	$[M\text{-}H]^- \rightarrow [M\text{-}H\text{-}H_3PO_2{}^{18}O_2]^-$	25	1
[$^{18}O_3$] ATP	512	408	$[M\text{-}H]^- \rightarrow [M\text{-}H\text{-}H_3PO^{18}O_3]^-$	25	1
[$^{18}O_2$] ATP	510	412	$[M\text{-}H]^- \rightarrow [M\text{-}H\text{-}H_3PO_4]^-$	25	1
[$^{18}O_2$] ATP	510	410	$[M\text{-}H]^- \rightarrow [M\text{-}H\text{-}H_3PO_3{}^{18}O]^-$	25	1
[$^{18}O_2$] ATP	510	408	$[M\text{-}H]^- \rightarrow [M\text{-}H\text{-}H_3PO_2{}^{18}O_2]^-$	25	1
[^{18}O] ATP	508	410	$[M\text{-}H]^- \rightarrow [M\text{-}H\text{-}H_3PO_3{}^{18}O]^-$	25	1
[^{18}O] ATP	508	408	$[M\text{-}H]^- \rightarrow [M\text{-}H\text{-}H_3PO_2{}^{18}O_2]^-$	25	1
ATP	506	408	$[M\text{-}H]^- \rightarrow [M\text{-}H\text{-}H_3PO_4]^-$	25	1
[$^{13}C_{10}$] ATP	516	418	$[M\text{-}H]^- \rightarrow [M\text{-}H\text{-}H_3PO_4]^-$	25	1

Notes: Negative polarity; fragmentor (V) is 166; dwell is 40. Unit or wide mass resolution was set accordingly.

Supplementary Table S4. MRM transitions setting of $InsP_{6-8}$ for the measurement of yeast and human cell samples

Molecular name	Precursor Ion	Product Ion	Type of transition	Collision Energy (V)	Cell Accelerator Voltage
[$^{13}C_6$] $InsP_8$	411.9	362.9	$[M-2H]^{2-} \rightarrow [M-2H-H_3PO_4]^{2-}$	10	1
$InsP_8$	408.9	359.9	$[M-2H]^{2-} \rightarrow [M-2H-H_3PO_4]^{2-}$	10	1
[^{18}O] $InsP_8$	409.9	360.9	$[M-2H]^{2-} \rightarrow [M-2H-H_3PO_4]^{2-}$	10	1
[^{18}O] $InsP_8$	409.9	359.9	$[M-2H]^{2-} \rightarrow [M-2H-H_3PO_3{}^{18}O]^{2-}$	10	1
[$^{18}O_2$] $InsP_8$	410.9	361.9	$[M-2H]^{2-} \rightarrow [M-2H-H_3PO_4]^{2-}$	10	1
[$^{18}O_2$] $InsP_8$	410.9	360.9	$[M-2H]^{2-} \rightarrow [M-2H-H_3PO_3{}^{18}O]^{2-}$	10	1
[$^{18}O_2$] $InsP_8$	410.9	359.9	$[M-2H]^{2-} \rightarrow [M-2H-H_3PO_2{}^{18}O_2]^{2-}$	10	1
[$^{13}C_6$] $InsP_7$	371.9	322.9	$[M-2H]^{2-} \rightarrow [M-2H-H_3PO_4]^{2-}$	10	3
$InsP_7$	368.9	319.9	$[M-2H]^{2-} \rightarrow [M-2H-H_3PO_4]^{2-}$	10	3
[^{18}O] $InsP_7$	369.9	320.9	$[M-2H]^{2-} \rightarrow [M-2H-H_3PO_4]^{2-}$	10	3
[^{18}O] $InsP_7$	369.9	319.9	$[M-2H]^{2-} \rightarrow [M-2H-H_3PO_3{}^{18}O]^{2-}$	10	3
[$^{18}O_2$] $InsP_7$	370.9	321.9	$[M-2H]^{2-} \rightarrow [M-2H-H_3PO_4]^{2-}$	10	3
[$^{18}O_2$] $InsP_7$	370.9	320.9	$[M-2H]^{2-} \rightarrow [M-2H-H_3PO_3{}^{18}O]^{2-}$	10	3
[$^{18}O_2$] $InsP_7$	370.9	319.9	$[M-2H]^{2-} \rightarrow [M-2H-H_3PO_2{}^{18}O_2]^{2-}$	10	3
[$^{13}C_6$] $InsP_6$	331.9	486.9	$[M-2H]^{2-} \rightarrow [M-H-HPO_3-H_3PO_4]^{-}$	13	4
$InsP_6$	328.9	480.9	$[M-2H]^{2-} \rightarrow [M-H-HPO_3-H_3PO_4]^{-}$	13	4
[^{18}O] $InsP_6$	329.9	482.9	$[M-2H]^{2-} \rightarrow [M-H-HPO_3-H_3PO_4]^{-}$	13	4
[^{18}O] $InsP_6$	329.9	480.9	$[M-2H]^{2-} \rightarrow [M-H-HPO_3- H_3PO_3{}^{18}O]^{-}$	13	4
[$^{18}O_2$] $InsP_6$	330.9	484.9	$[M-2H]^{2-} \rightarrow [M-H-HPO_3-H_3PO_4]^{-}$	13	4
[$^{18}O_2$] $InsP_6$	330.9	482.9	$[M-2H]^{2-} \rightarrow [M-H-HPO_3-H_3PO_3{}^{18}O]^{-}$	13	4
[$^{18}O_2$] $InsP_6$	330.9	480.9	$[M-2H]^{2-} \rightarrow [M-H-HPO_3-H_3PO_2{}^{18}O_2]^{-}$	13	4

Notes: Negative polarity; fragmentor (V) is 166; dwell is 80. Unit or wide mass resolution was applied accordingly.

Supplementary Table S5. MRM transitions setting of $InsP_{3-5}$ for the measurement of yeast and human cell samples

Molecular name	Precursor Ion	Product Ion	Type of transition	Collision Energy (V)	Cell Accelerator Voltage
$[^{13}C_6]$ $InsP_5$	292	504.9	$[M\text{-}2H]^{2-} \rightarrow [M\text{-}H\text{-}HPO_3]^-$	9	3
$InsP_5$	289	498.9	$[M\text{-}2H]^{2-} \rightarrow [M\text{-}H\text{-}HPO_3]^-$	9	3
$[^{18}O]$ $InsP_5$	290	500.9	$[M\text{-}2H]^{2-} \rightarrow [M\text{-}H\text{-}HPO_3]^-$	9	3
$[^{18}O]$ $InsP_5$	290	498.9	$[M\text{-}2H]^{2-} \rightarrow [M\text{-}H\text{-}HPO_2{}^{18}O]^-$	9	3
$[^{18}O_2]$ $InsP_5$	291	502.9	$[M\text{-}2H]^{2-} \rightarrow [M\text{-}H\text{-}HPO_3]^-$	9	3
$[^{18}O_2]$ $InsP_5$	291	500.9	$[M\text{-}2H]^{2-} \rightarrow [M\text{-}H\text{-}HPO_2{}^{18}O]^-$	9	3
$[^{18}O_2]$ $InsP_5$	291	498.9	$[M\text{-}2H]^{2-} \rightarrow [M\text{-}H\text{-}HPO^{18}O_2]^-$	9	3
$InsP_4$	249	418.9	$[M\text{-}2H]^{2-} \rightarrow [M\text{-}H\text{-}HPO_3]^-$	5	1
$[^{18}O]$ $InsP_4$	250	420.9	$[M\text{-}2H]^{2-} \rightarrow [M\text{-}H\text{-}HPO_3]^-$	10	3
$[^{18}O]$ $InsP_4$	250	418.9	$[M\text{-}2H]^{2-} \rightarrow [M\text{-}H\text{-}HPO_2{}^{18}O]^-$	10	3
$[^{18}O_2]$ $InsP_4$	251	422.9	$[M\text{-}2H]^{2-} \rightarrow [M\text{-}H\text{-}HPO_3]^-$	10	3
$[^{18}O_2]$ $InsP_4$	251	420.9	$[M\text{-}2H]^{2-} \rightarrow [M\text{-}H\text{-}HPO_2{}^{18}O]^-$	10	3
$[^{18}O_2]$ $InsP_4$	251	418.9	$[M\text{-}2H]^{2-} \rightarrow [M\text{-}H\text{-}HPO^{18}O_2]^-$	10	3
$InsP_3$	418.9	320.8	$[M\text{-}H]^- \rightarrow [M\text{-}H\text{-}H_3PO_4]^-$	17	4
$[^{18}O]$ $InsP_3$	420.9	322.8	$[M\text{-}H]^- \rightarrow [M\text{-}H\text{-}H_3PO_4]^-$	17	4
$[^{18}O]$ $InsP_3$	420.9	320.8	$[M\text{-}H]^- \rightarrow [M\text{-}H\text{-}H_3PO_3{}^{18}O]^-$	17	4
$[^{18}O_2]$ $InsP_3$	422.9	324.9	$[M\text{-}H]^- \rightarrow [M\text{-}H\text{-}H_3PO_4]^-$	17	4
$[^{18}O_2]$ $InsP_3$	422.9	322.9	$[M\text{-}H]^- \rightarrow [M\text{-}H\text{-}H_3PO_3{}^{18}O]^-$	17	4
$[^{18}O_2]$ $InsP_3$	422.9	320.9	$[M\text{-}H]^- \rightarrow [M\text{-}H\text{-}H_3PO_2{}^{18}O_2]^-$	17	4

Notes: Negative polarity; fragmentor (V) is 166; dwell is 60. Wide mass resolution was applied.

Supplementary Table S6. MRM transitions setting of $InsP_8$ for amoeba samples

Molecular name	Precursor Ion	Product Ion	Type of transition	Collision Energy (V)	Cell Accelerator Voltage
$InsP_8$	408.9	359.9	$[M-2H]^{2-} \rightarrow [M-2H-H_3PO_4]^{2-}$	10	1
$[^{18}O]\ InsP_8$	409.9	360.9	$[M-2H]^{2-} \rightarrow [M-2H-H_3PO_4]^{2-}$	10	1
$[^{18}O]\ InsP_8$	409.9	359.9	$[M-2H]^{2-} \rightarrow [M-2H-H_3PO_3{}^{18}O]^{2-}$	10	1
$[^{18}O_2]\ InsP_8$	410.9	361.9	$[M-2H]^{2-} \rightarrow [M-2H-H_3PO_4]^{2-}$	10	1
$[^{18}O_2]\ InsP_8$	410.9	360.9	$[M-2H]^{2-} \rightarrow [M-2H-H_3PO_3{}^{18}O]^{2-}$	10	1
$[^{18}O_2]\ InsP_8$	410.9	359.9	$[M-2H]^{2-} \rightarrow [M-2H-H_3PO_2{}^{18}O_2]^{2-}$	10	1
$[^{18}O_3]\ InsP_8$	411.9	362.9	$[M-2H]^{2-} \rightarrow [M-2H-H_3PO_4]^{2-}$	10	1
$[^{18}O_3]\ InsP_8$	411.9	361.9	$[M-2H]^{2-} \rightarrow [M-2H-H_3PO_3{}^{18}O]^{2-}$	10	1
$[^{18}O_3]\ InsP_8$	411.9	360.9	$[M-2H]^{2-} \rightarrow [M-2H-H_3PO_2{}^{18}O_2]^{2-}$	10	1
$[^{18}O_3]\ InsP_8$	411.9	359.9	$[M-2H]^{2-} \rightarrow [M-2H-H_3PO^{18}O_3]^{2-}$	10	1
$[^{18}O_4]\ InsP_8$	412.9	363.9	$[M-2H]^{2-} \rightarrow [M-2H-H_3PO_4]^{2-}$	10	1
$[^{18}O_4]\ InsP_8$	412.9	362.9	$[M-2H]^{2-} \rightarrow [M-2H-H_3PO_3{}^{18}O]^{2-}$	10	1
$[^{18}O_4]\ InsP_8$	412.9	361.9	$[M-2H]^{2-} \rightarrow [M-2H-H_3PO_2{}^{18}O_2]^{2-}$	10	1
$[^{18}O_4]\ InsP_8$	412.9	360.9	$[M-2H]^{2-} \rightarrow [M-2H-H_3PO^{18}O_3]^{2-}$	10	1
$[^{18}O_4]\ InsP_8$	412.9	359.9	$[M-2H]^{2-} \rightarrow [M-2H-H_3P^{18}O_4]^{2-}$	10	1
$[^{18}O_5]\ InsP_8$	413.9	364.9	$[M-2H]^{2-} \rightarrow [M-2H-H_3PO_4]^{2-}$	10	1
$[^{18}O_5]\ InsP_8$	413.9	363.9	$[M-2H]^{2-} \rightarrow [M-2H-H_3PO_3{}^{18}O]^{2-}$	10	1
$[^{18}O_5]\ InsP_8$	413.9	362.9	$[M-2H]^{2-} \rightarrow [M-2H-H_3PO_2{}^{18}O_2]^{2-}$	10	1
$[^{18}O_5]\ InsP_8$	413.9	361.9	$[M-2H]^{2-} \rightarrow [M-2H-H_3PO^{18}O_3]^{2-}$	10	1
$[^{18}O_5]\ InsP_8$	413.9	360.9	$[M-2H]^{2-} \rightarrow [M-2H-H_3P^{18}O_4]^{2-}$	10	1
$[^{18}O_6]\ InsP_8$	414.9	365.9	$[M-2H]^{2-} \rightarrow [M-2H-H_3PO_4]^{2-}$	10	1
$[^{18}O_6]\ InsP_8$	414.9	364.9	$[M-2H]^{2-} \rightarrow [M-2H-H_3PO_3{}^{18}O]^{2-}$	10	1
$[^{18}O_6]\ InsP_8$	414.9	363.9	$[M-2H]^{2-} \rightarrow [M-2H-H_3PO_2{}^{18}O_2]^{2-}$	10	1
$[^{18}O_6]\ InsP_8$	414.9	362.9	$[M-2H]^{2-} \rightarrow [M-2H-H_3PO^{18}O_3]^{2-}$	10	1
$[^{18}O_6]\ InsP_8$	414.9	361.9	$[M-2H]^{2-} \rightarrow [M-2H-H_3P^{18}O_4]^{2-}$	10	1

Notes: Negative polarity; fragmentor (V) is 166; dwell is 25. Wide mass resolution was applied.

Supplementary Table S7. MRM transitions setting of $InsP_7$ for amoeba samples

Molecular name	Precursor Ion	Product Ion	Type of transition	Collision Energy (V)	Cell Accelerator Voltage
$InsP_7$	368.9	319.9	$[M-2H]^{2-} \rightarrow [M-2H-H_3PO_4]^{2-}$	10	3
$[^{18}O]$ $InsP_7$	369.9	320.9	$[M-2H]^{2-} \rightarrow [M-2H-H_3PO_4]^{2-}$	10	3
$[^{18}O]$ $InsP_7$	369.9	319.9	$[M-2H]^{2-} \rightarrow [M-2H-H_3PO_3{}^{18}O]^{2-}$	10	3
$[^{18}O_2]$ $InsP_7$	370.9	321.9	$[M-2H]^{2-} \rightarrow [M-2H-H_3PO_4]^{2-}$	10	3
$[^{18}O_2]$ $InsP_7$	370.9	320.9	$[M-2H]^{2-} \rightarrow [M-2H-H_3PO_3{}^{18}O]^{2-}$	10	3
$[^{18}O_2]$ $InsP_7$	370.9	319.9	$[M-2H]^{2-} \rightarrow [M-2H-H_3PO_2{}^{18}O_2]^{2-}$	10	3
$[^{18}O_3]$ $InsP_7$	371.9	322.9	$[M-2H]^{2-} \rightarrow [M-2H-H_3PO_4]^{2-}$	10	3
$[^{18}O_3]$ $InsP_7$	371.9	321.9	$[M-2H]^{2-} \rightarrow [M-2H-H_3PO_3{}^{18}O]^{2-}$	10	3
$[^{18}O_3]$ $InsP_7$	371.9	320.9	$[M-2H]^{2-} \rightarrow [M-2H-H_3PO_2{}^{18}O_2]^{2-}$	10	3
$[^{18}O_3]$ $InsP_7$	371.9	319.9	$[M-2H]^{2-} \rightarrow [M-2H-H_3PO^{18}O_3]^{2-}$	10	3
$[^{18}O_4]$ $InsP_7$	372.9	323.9	$[M-2H]^{2-} \rightarrow [M-2H-H_3PO_4]^{2-}$	10	3
$[^{18}O_4]$ $InsP_7$	372.9	322.9	$[M-2H]^{2-} \rightarrow [M-2H-H_3PO_3{}^{18}O]^{2-}$	10	3
$[^{18}O_4]$ $InsP_7$	372.9	321.9	$[M-2H]^{2-} \rightarrow [M-2H-H_3PO_2{}^{18}O_2]^{2-}$	10	3
$[^{18}O_4]$ $InsP_7$	372.9	320.9	$[M-2H]^{2-} \rightarrow [M-2H-H_3PO^{18}O_3]^{2-}$	10	3
$[^{18}O_4]$ $InsP_7$	372.9	319.9	$[M-2H]^{2-} \rightarrow [M-2H-H_3P^{18}O_4]^{2-}$	10	3
$[^{18}O_5]$ $InsP_7$	373.9	324.9	$[M-2H]^{2-} \rightarrow [M-2H-H_3PO_4]^{2-}$	10	3
$[^{18}O_5]$ $InsP_7$	373.9	323.9	$[M-2H]^{2-} \rightarrow [M-2H-H_3PO_3{}^{18}O]^{2-}$	10	3
$[^{18}O_5]$ $InsP_7$	373.9	322.9	$[M-2H]^{2-} \rightarrow [M-2H-H_3PO_2{}^{18}O_2]^{2-}$	10	3
$[^{18}O_5]$ $InsP_7$	373.9	321.9	$[M-2H]^{2-} \rightarrow [M-2H-H_3PO^{18}O_3]^{2-}$	10	3
$[^{18}O_5]$ $InsP_7$	373.9	320.9	$[M-2H]^{2-} \rightarrow [M-2H-H_3P^{18}O_4]^{2-}$	10	3
$[^{18}O_6]$ $InsP_7$	374.9	325.9	$[M-2H]^{2-} \rightarrow [M-2H-H_3PO_4]^{2-}$	10	3
$[^{18}O_6]$ $InsP_7$	374.9	324.9	$[M-2H]^{2-} \rightarrow [M-2H-H_3PO_3{}^{18}O]^{2-}$	10	3
$[^{18}O_6]$ $InsP_7$	374.9	323.9	$[M-2H]^{2-} \rightarrow [M-2H-H_3PO_2{}^{18}O_2]^{2-}$	10	3
$[^{18}O_6]$ $InsP_7$	374.9	322.9	$[M-2H]^{2-} \rightarrow [M-2H-H_3PO^{18}O_3]^{2-}$	10	3
$[^{18}O_6]$ $InsP_7$	374.9	321.9	$[M-2H]^{2-} \rightarrow [M-2H-H_3P^{18}O_4]^{2-}$	10	3

Notes: Negative polarity; fragmentor (V) is 166; dwell is 25. Wide mass resolution was applied.

Supplementary Table S8. MRM transitions setting of $InsP_6$ for amoeba samples

Molecular name	Precursor Ion	Product Ion	Type of transition	Collision Energy (V)	Cell Accelerator Voltage
$InsP_6$	328.9	79	$[M-2H]^{2-} \rightarrow [PO_3]^-$	13	4
$[^{18}O]$ $InsP_6$	329.9	81	$[M-2H]^{2-} \rightarrow [PO_2{}^{18}O]^-$	13	4
$[^{18}O]$ $InsP_6$	329.9	79	$[M-2H]^{2-} \rightarrow [PO_3]^-$	13	4
$[^{18}O_2]$ $InsP_6$	330.9	83	$[M-2H]^{2-} \rightarrow [PO^{18}O_2]^-$	13	4
$[^{18}O_2]$ $InsP_6$	330.9	81	$[M-2H]^{2-} \rightarrow [PO_2{}^{18}O]^-$	13	4
$[^{18}O_2]$ $InsP_6$	330.9	79	$[M-2H]^{2-} \rightarrow [PO_3]^-$	13	4
$[^{18}O_3]$ $InsP_6$	331.9	85	$[M-2H]^{2-} \rightarrow [P^{18}O_3]^-$	13	4
$[^{18}O_3]$ $InsP_6$	331.9	83	$[M-2H]^{2-} \rightarrow [PO^{18}O_2]^-$	13	4
$[^{18}O_3]$ $InsP_6$	331.9	81	$[M-2H]^{2-} \rightarrow [PO_2{}^{18}O]^-$	13	4
$[^{18}O_3]$ $InsP_6$	331.9	79	$[M-2H]^{2-} \rightarrow [PO_3]^-$	13	4
$[^{18}O_4]$ $InsP_6$	332.9	85	$[M-2H]^{2-} \rightarrow [P^{18}O_3]^-$	13	4
$[^{18}O_4]$ $InsP_6$	332.9	83	$[M-2H]^{2-} \rightarrow [PO^{18}O_2]^-$	13	4
$[^{18}O_4]$ $InsP_6$	332.9	81	$[M-2H]^{2-} \rightarrow [PO_2{}^{18}O]^-$	13	4
$[^{18}O_4]$ $InsP_6$	332.9	79	$[M-2H]^{2-} \rightarrow [PO_3]^-$	13	4
$[^{18}O_5]$ $InsP_6$	333.9	85	$[M-2H]^{2-} \rightarrow [P^{18}O_3]^-$	13	4
$[^{18}O_5]$ $InsP_6$	333.9	83	$[M-2H]^{2-} \rightarrow [PO^{18}O_2]^-$	13	4
$[^{18}O_5]$ $InsP_6$	333.9	81	$[M-2H]^{2-} \rightarrow [PO_2{}^{18}O]^-$	13	4
$[^{18}O_5]$ $InsP_6$	333.9	79	$[M-2H]^{2-} \rightarrow [PO_3]^-$	13	4
$[^{18}O_6]$ $InsP_6$	334.9	85	$[M-2H]^{2-} \rightarrow [P^{18}O_3]^-$	13	4
$[^{18}O_6]$ $InsP_6$	334.9	83	$[M-2H]^{2-} \rightarrow [PO^{18}O_2]^-$	13	4
$[^{18}O_6]$ $InsP_6$	334.9	81	$[M-2H]^{2-} \rightarrow [PO_2{}^{18}O]^-$	13	4
$[^{18}O_6]$ $InsP_6$	334.9	79	$[M-2H]^{2-} \rightarrow [PO_3]^-$	13	4
$[^{18}O_7]$ $InsP_6$	335.9	85	$[M-2H]^{2-} \rightarrow [P^{18}O_3]^-$	13	4
$[^{18}O_7]$ $InsP_6$	335.9	83	$[M-2H]^{2-} \rightarrow [PO^{18}O_2]^-$	13	4
$[^{18}O_7]$ $InsP_6$	335.9	81	$[M-2H]^{2-} \rightarrow [PO_2{}^{18}O]^-$	13	4
$[^{18}O_7]$ $InsP_6$	335.9	79	$[M-2H]^{2-} \rightarrow [PO_3]^-$	13	4
$[^{18}O_8]$ $InsP_6$	336.9	85	$[M-2H]^{2-} \rightarrow [P^{18}O_3]^-$	13	4
$[^{18}O_8]$ $InsP_6$	336.9	83	$[M-2H]^{2-} \rightarrow [PO^{18}O_2]^-$	13	4
$[^{18}O_8]$ $InsP_6$	336.9	81	$[M-2H]^{2-} \rightarrow [PO_2{}^{18}O]^-$	13	4
$[^{18}O_8]$ $InsP_6$	336.9	79	$[M-2H]^{2-} \rightarrow [PO_3]^-$	13	4

Notes: Negative polarity; fragmentor (V) is 166; dwell is 30. Wide mass resolution was applied.

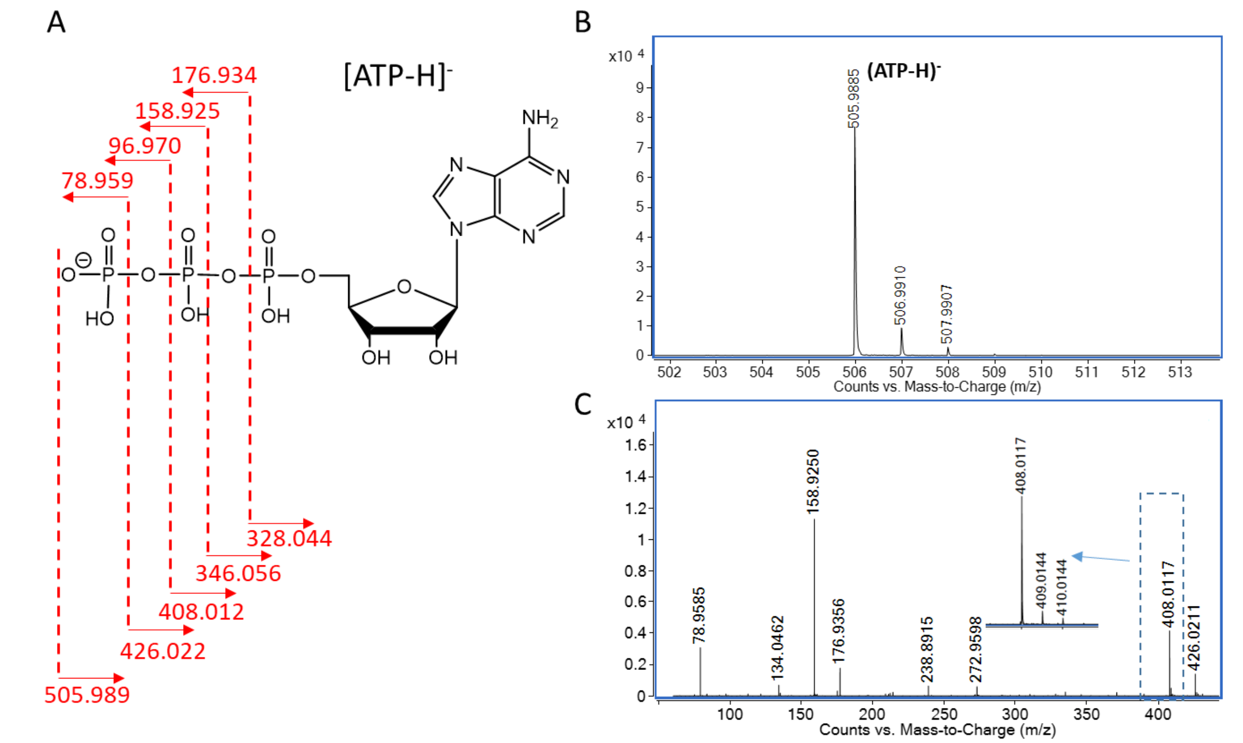

Supplementary Figure S1. ESI-MS analysis of ATP ESI-MS fragmentations. **A** Structure and proposed ESI-MS fragmentations of ATP. **B** ESI-MS of ATP standard. **C** Observed ESI-MS fragmentations of ATP by qTOF. 408.0117, which corresponds to (ATP-H-H_3PO_4)$^-$, was taken as the special product ion for the QQQ analysis. The ion at *m/z* 408.0117 was also the maximized specific product ion signal by MassHunter optimizer with injection of standard.

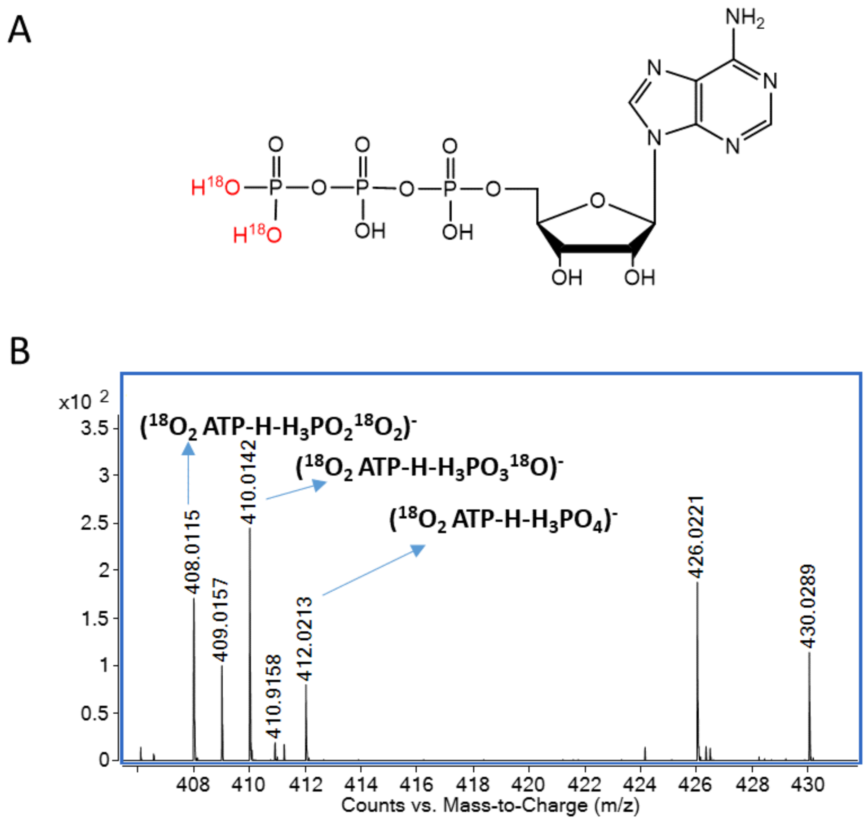

Supplementary Figure S2. Oxygen migration (scrambling) on ATP in the gas phase. **A** Structure of synthetic γ-$^{18}O_2$ labeled ATP. **B** ($^{18}O_2$ ATP-H-$H_3PO_2{}^{18}O_2$)$^-$ (34.8%), ($^{18}O_2$ ATP-H-$H_3PO_3{}^{18}O$)$^-$ (49%) and ($^{18}O_2$ ATP-H-H_3PO_4)$^-$ (16%) were observed, indicating partial scrambling of ^{18}O in the gas phase (qTOF).

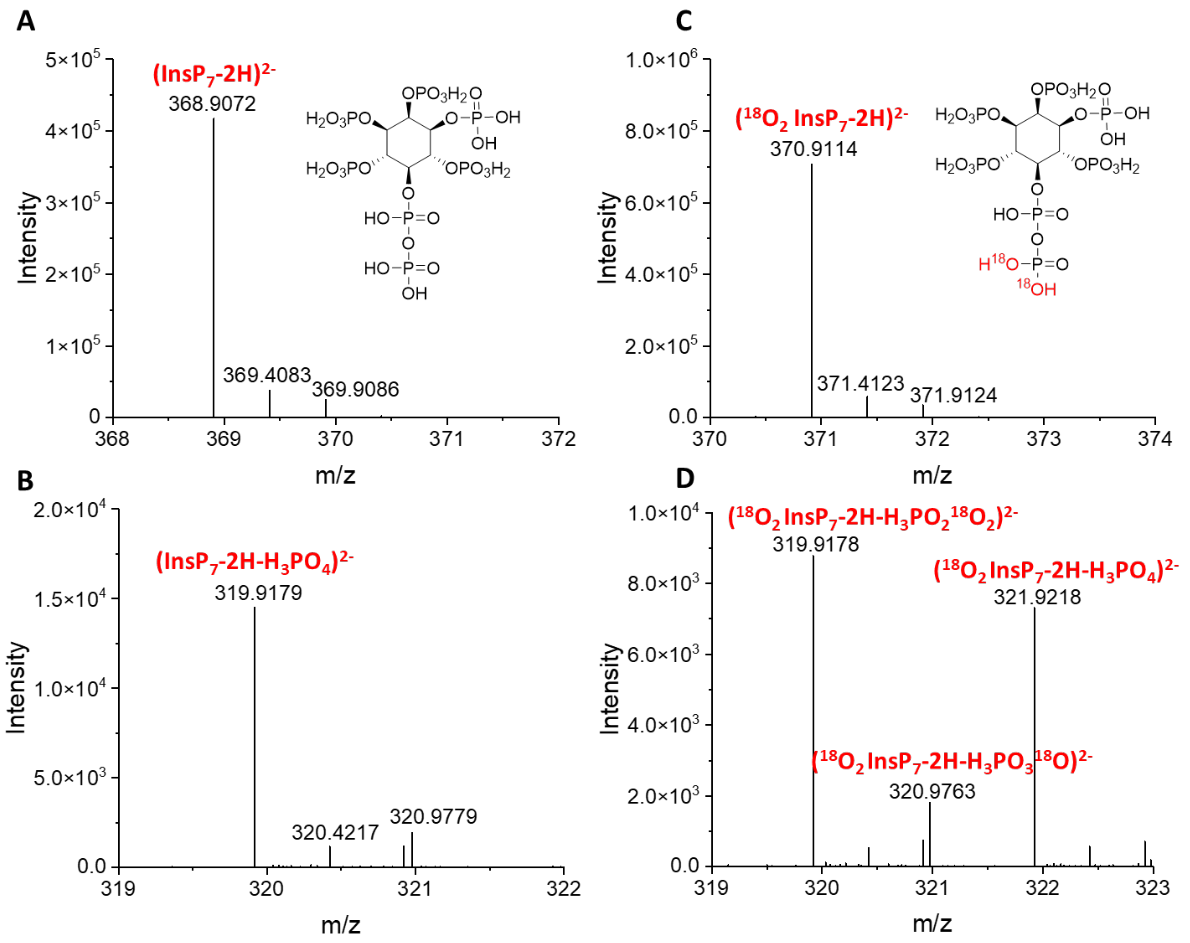

Supplementary Figure S3. Oxygen migration (scrambling) on 5-$InsP_7$ in the gas phase. **A** ESI-MS of 5-$InsP_7$ standard. **B** Observed ESI-MS fragmentations of 5-$InsP_7$ standard by qTOF. **C** ESI-MS of synthetic $^{18}O_2$ 5-$InsP_7$ standard. **D** Observed ESI-MS fragmentations of synthetic $^{18}O_2$ 5-$InsP_7$ standard by qTOF. ($^{18}O_2$ 5-$InsP_7$-2H-$H_3PO_2{}^{18}O_2)^{2-}$ (49%), ($^{18}O_2$ 5-$InsP_7$-2H-$H_3PO_3{}^{18}O)^{2-}$ (10%) and ($^{18}O_2$ 5-$InsP_7$-2H-$H_3PO_4)^{2-}$ (41%) were observed, indicating partial scrambling of ^{18}O in the gas phase is also occurring in $InsP_7$.

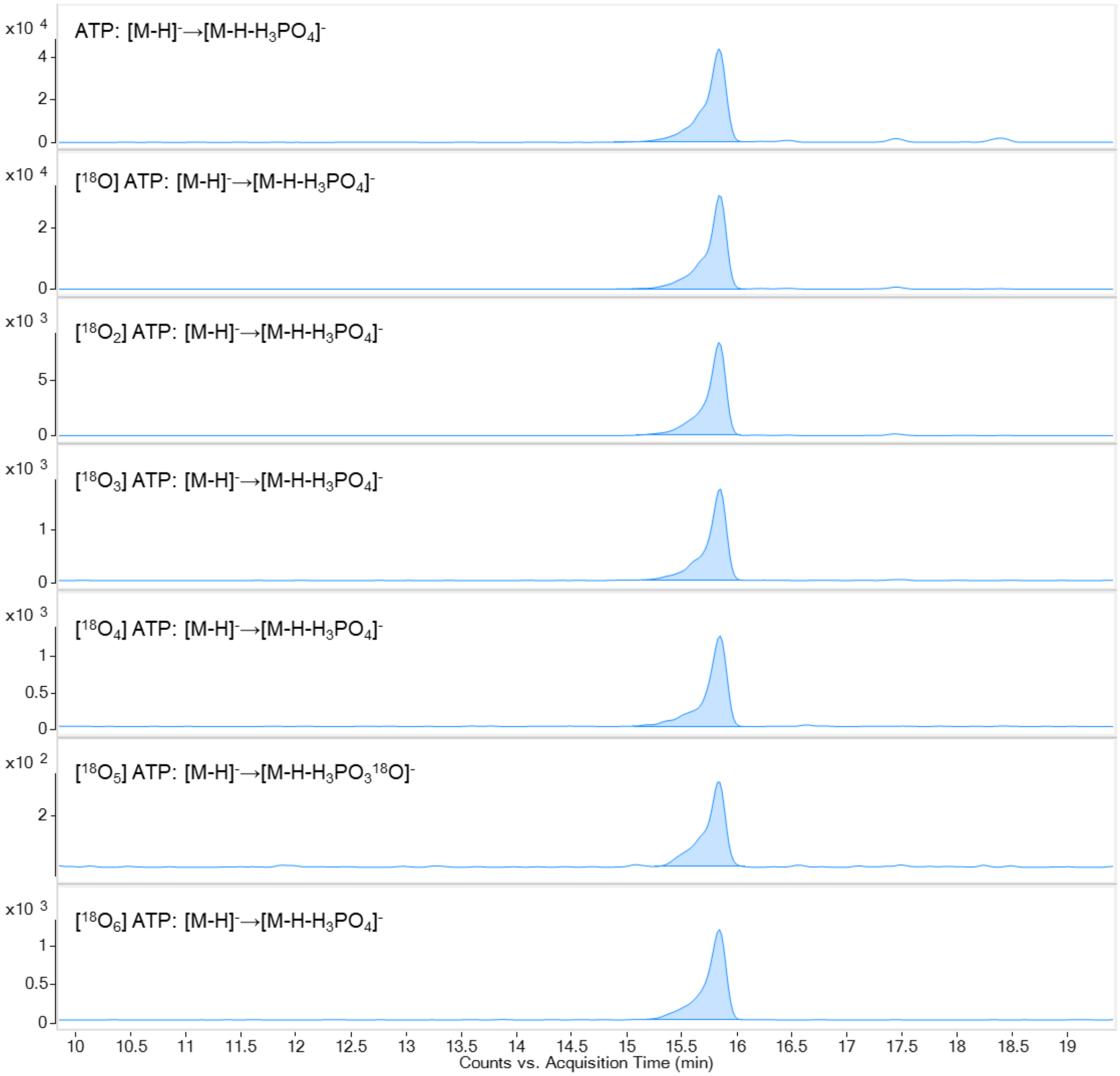

Supplementary Figure S4. Extracted ion electropherograms (EIEs) of unlabeled and ^{18}O labeled ATP from yeast under steady state conditions at the 1 min time point. (CE-QQQ)

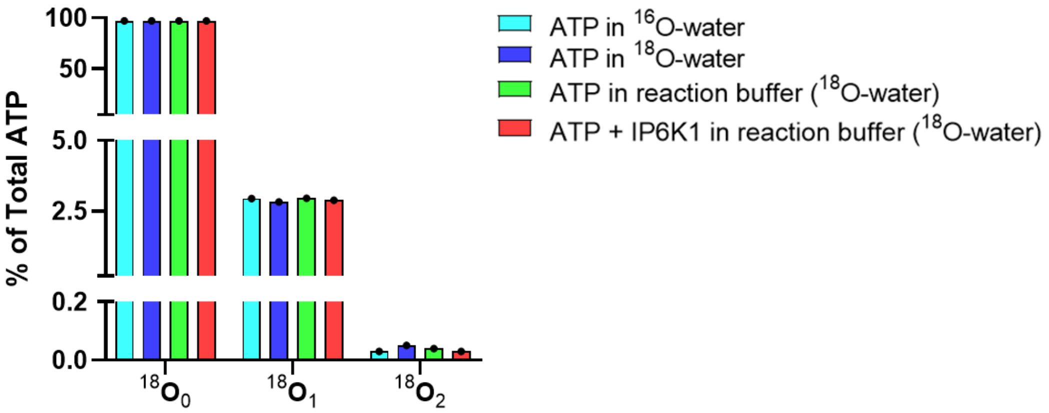

	ATP in ^{16}O-water	ATP in ^{18}O-water	ATP in reaction buffer (^{18}O-water)	ATP + IP6K1 in reaction buffer (^{18}O-water)
water	^{16}O-water	50% of ^{18}O-water	50% of ^{18}O-water	50% of ^{18}O-water
Reaction buffer	No	No	Yes	Yes
ATP	1 mM	1 mM	1 mM	1 mM
IP6K1	No	No	No	Yes

Supplementary Figure S5. ATP does not undergo spontaneous $^{16/18}$ oxygen exchange in ^{18}O-water or with inositol hexakisphosphate kinase IP6K1 after 4 hours. Samples were measured by CE-QQQ with unit mass resolution method.

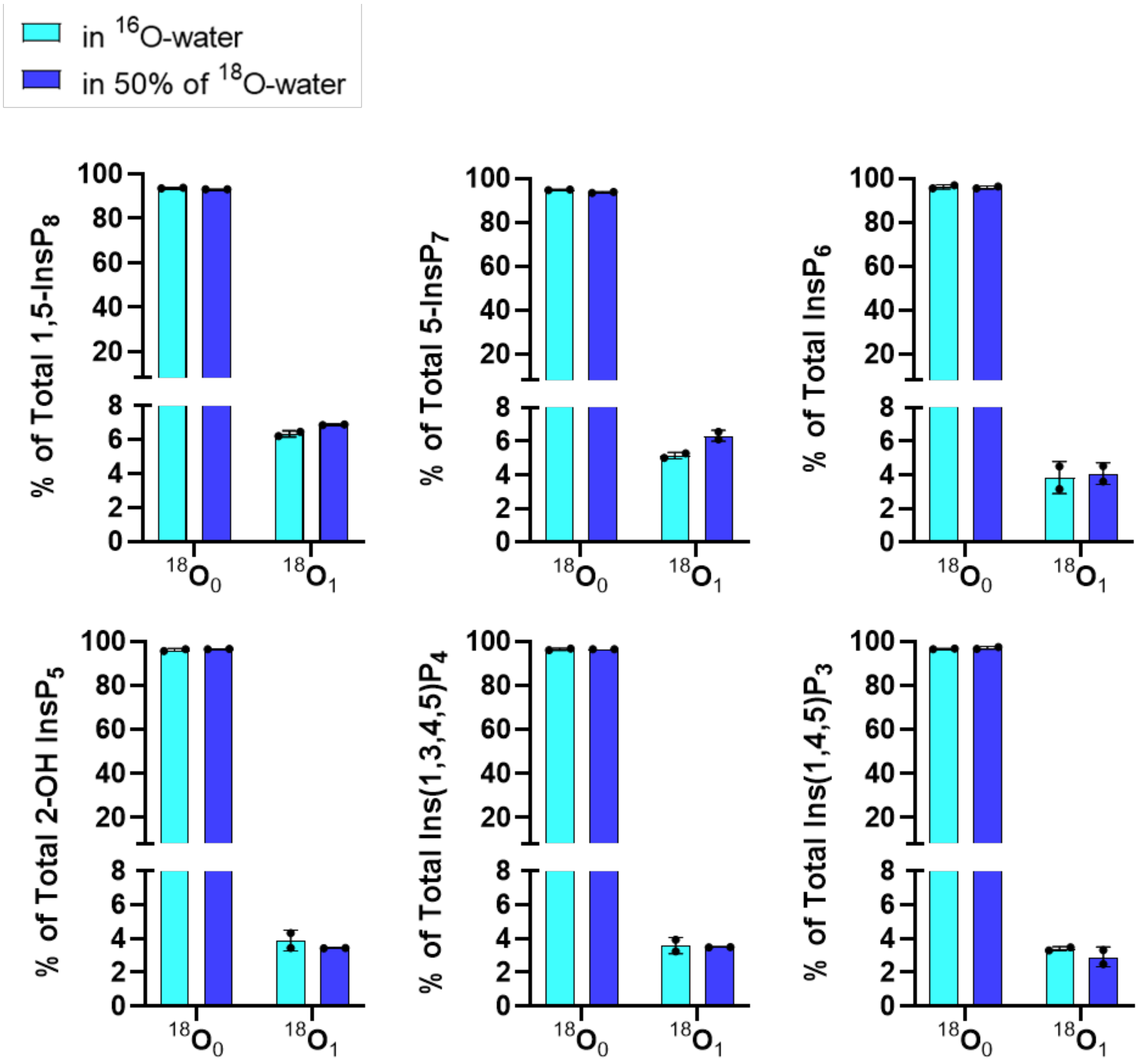

Supplementary Figure S6. Ins(1,4,5)P_3, Ins(1,3,4,5)P_4, 2-OH InsP_5, InsP_6, 5-InsP_7 and 1,5-InsP_8 do not undergo spontaneous 16/18 oxygen exchange in the presence of 50% of ^{18}O-water (>98 atom% ^{18}O) after 4 hours. The means of two samples with standard deviation are shown. Samples were measured by CE-QQQ with unit mass resolution method.

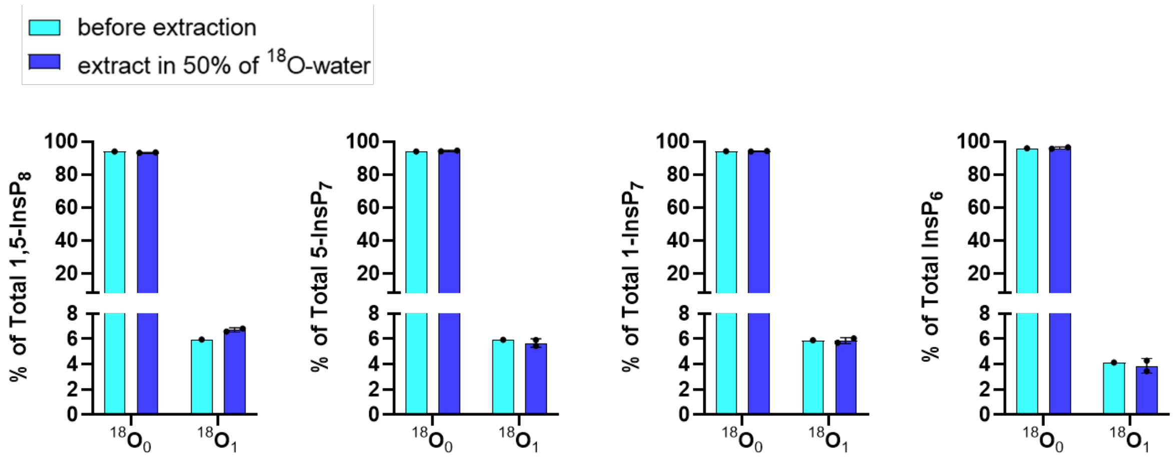

Supplementary Figure S7. $InsP_6$, 5-$InsP_7$, 1-$InsP_7$, and 1,5-$InsP_8$ do not undergo spontaneous $^{16/18}$ oxygen exchange when incubating InsPs and PP-InsPs with perchloric acid containing 50% of ^{18}O-labeling water (>98 atom% ^{18}O) and subjected to TiO_2 purification. The means of two samples with standard deviation are shown for the extracted samples in ^{18}O-water. One sample result is shown for control sample (before extraction). Samples were measured by CE-QQQ with unit mass resolution method.

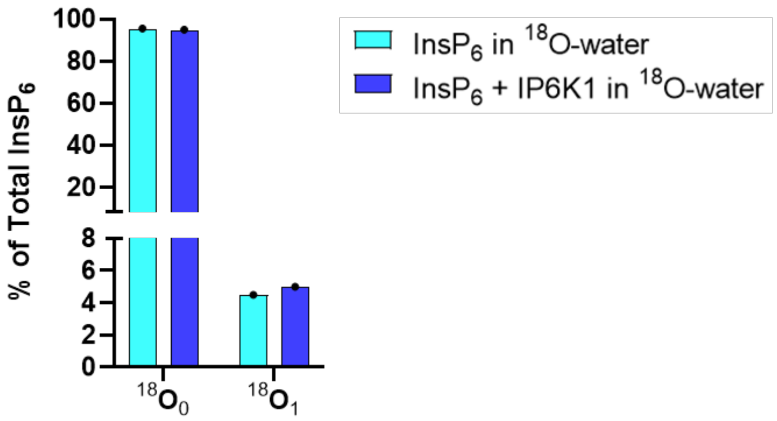

	$InsP_6$ in ^{18}O-water	$InsP_6$ + IP6K1 in ^{18}O-water
water	50% of ^{18}O-water	50% of ^{18}O-water
Reaction buffer	Yes	Yes
$InsP_6$	100 µM	100 µM
IP6K1	No	Yes

Supplementary Figure S8. $InsP_6$ does not undergo spontaneous $^{16/18}$ oxygen exchange in ^{18}O-water or in presence of inositol hexakisphosphate kinase IP6K1 after 4 hours. Samples were measured by CE-QQQ with unit mass resolution method.

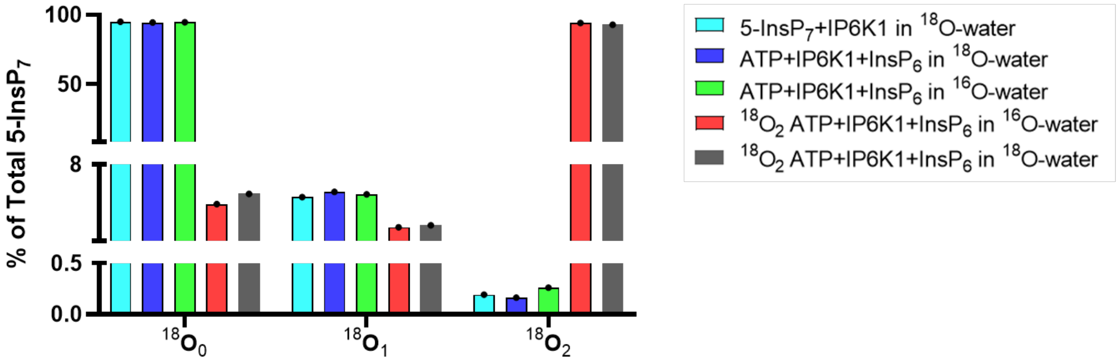

	5-$InsP_7$+IP6K1 in ^{18}O-water	ATP+IP6K1+$InsP_6$ ^{18}O-water	ATP+IP6K1+$InsP_6$ in ^{16}O-water	$^{18}O_2$ ATP+IP6K1+$InsP_6$ in ^{16}O-water	$^{18}O_2$ ATP+IP6K1+$InsP_6$ in ^{18}O-water
water	50% of ^{18}O-water	50% of ^{18}O-water	^{16}O-water	^{16}O-water	50% of ^{18}O-water
Reaction buffer	Yes	Yes	Yes	Yes	Yes
5-$InsP_7$	100 μM	No	No	No	No
$InsP_6$	No	100 μM	100 μM	100 μM	100 μM
ATP	No	1 mM	1 mM	No	No
$^{18}O_2$ ATP	No	No	No	1 mM	1 mM
IP6K1	Yes	Yes	Yes	Yes	Yes

Supplementary Figure S9. 5-$InsP_7$ does not undergo spontaneous $^{16/18}$ oxygen exchange in ^{18}O-water and inositol hexakisphosphate kinase IP6K1 after 4 hours. The results of IP6K1 in vitro assays shown no ^{18}O labeled 5-$InsP_7$ generated in the presence of ^{18}O-water; however, $^{18}O_2$ labeled 5-$InsP_7$ was produced when $^{18}O_2$ ATP was used. Samples were measured by CE-QQQ with unit mass resolution method.

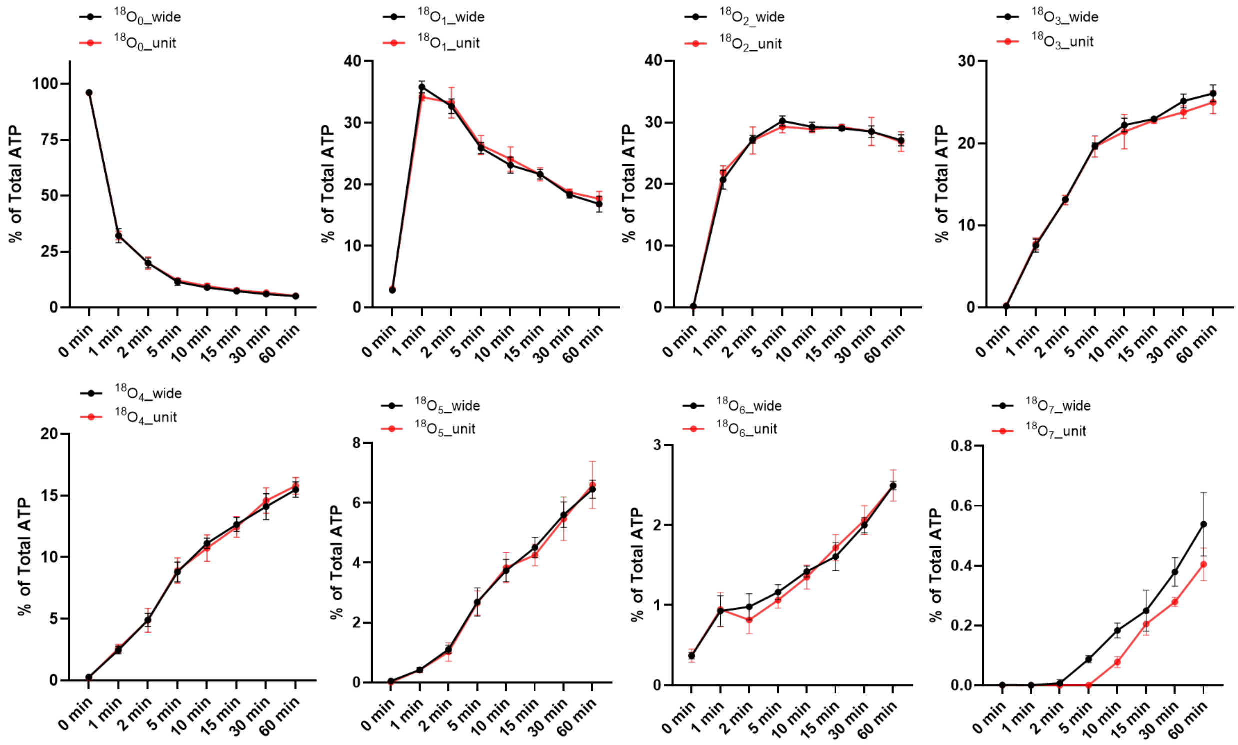

Supplementary Figure S10. 50% of ^{18}O-labelled water experiment: time-dependent formation of ATP isotopologues in yeast with different numbers of ^{18}O atoms studied by CE-QQQ wide mass resolution method and unit resolution method, respectively. The means of 3 samples with standard deviation are shown. Wild type yeast cells were grown logarithmically in SC medium at 20˚C. At the 0 min time point, the medium was changed to SC medium prepared with 50% of ^{18}O-labelled water (99% enrichment).

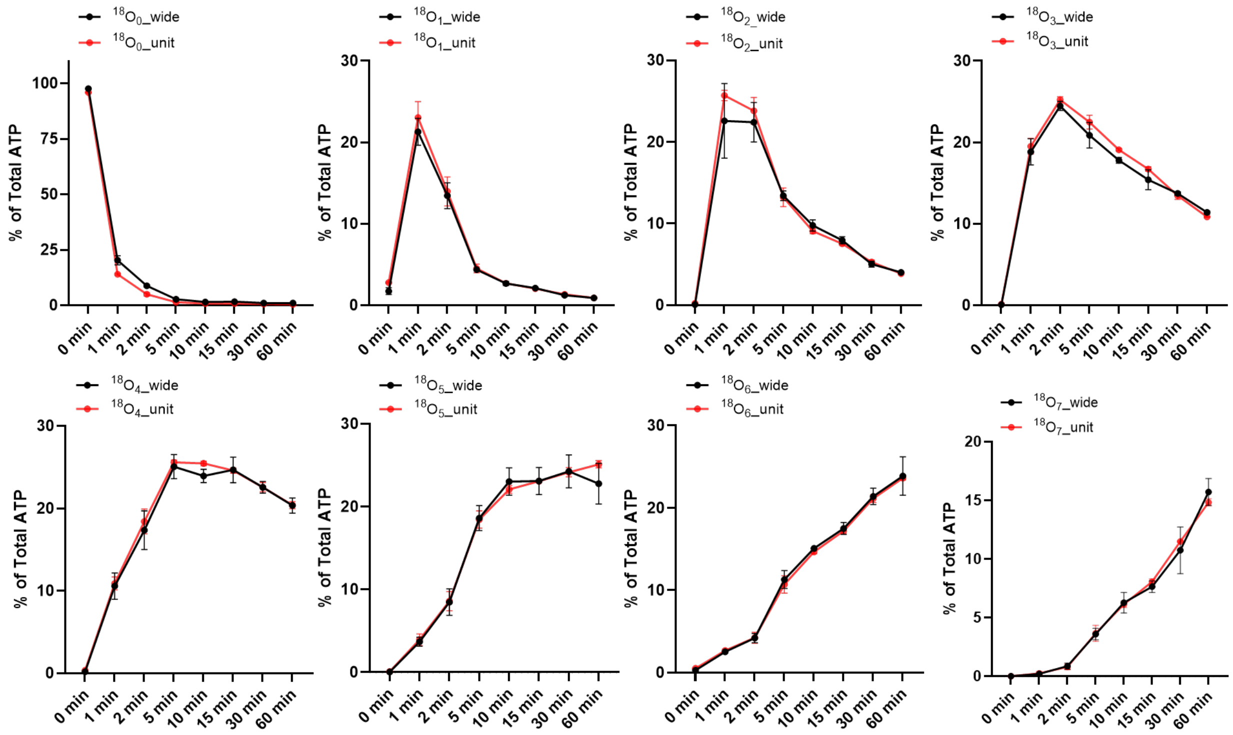

Supplementary Figure S11. 100% of ^{18}O-labelled water experiment: time-dependent formation of ATP isotopologues in yeast with different numbers of ^{18}O atoms studied by CE-QQQ wide mass resolution method and unit resolution method, respectively. The means of 3 samples with standard deviation are shown. Wild type yeast cells were grown logarithmically in SC medium at 20˚C. At the 0 min time point, the medium was changed to SC medium prepared with 100% of ^{18}O-labelled water (99% enrichment).

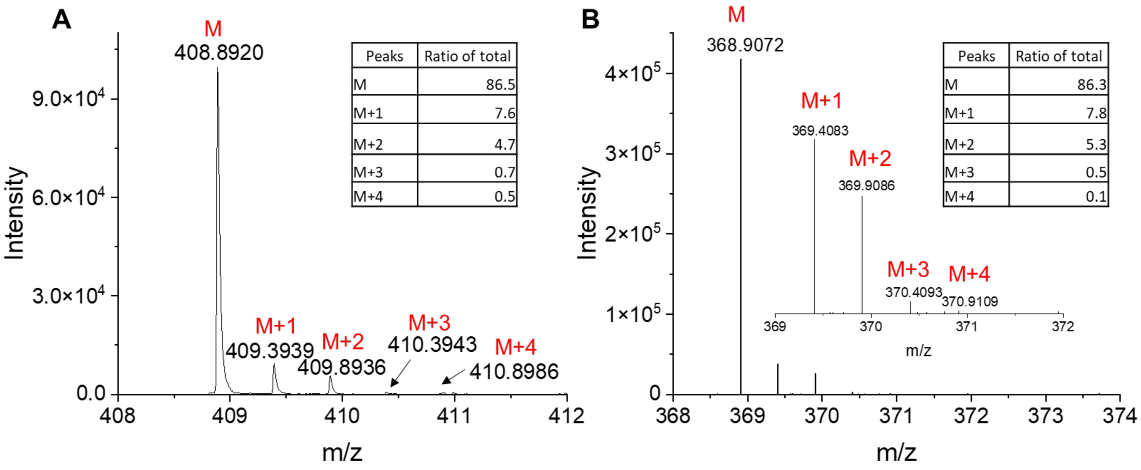

C

	Wide mass resolution (±0.6 Da mass error)			Unit mass resolution (±0.35 Da mass error)		
	% of total			% of total		
	$^{18}O_0$ (M)	$^{18}O_1$ (M+2)	$^{18}O_2$ (M+4)	$^{18}O_0$ (M)	$^{18}O_1$ (M+2)	$^{18}O_2$ (M+4)
1,5-InsP_8	87.9	11.6	0.5	94.0	5.8	0.2
5-InsP_7	88.4	11.2	0.4	94.4	5.6	0.04

Supplementary Figure S12. Effect of the isotopic peak. **A** Distribution of isotopic species in unlabeled 1,5-InsP_8 was detected by qTOF in the state of double charged ion. **B** Distribution of isotopic species in unlabeled 5-InsP_7 was detected by qTOF in the status of double charged ion. Thus, the delta mass between M and M+1 is 0.5 Da. **C** Unlabeled 1,5-InsP_8 and 5-InsP_7 were detected by CE-QQQ system with both wide mass resolution and unit mass resolution. Unit mass resolution is able to resolve M, M+1, M+2, M+3, and M+4, because the unit mass error is ± 0.35 Da, which is narrower than the delta mass of doubly charged isotopic species (0.5 Da). Nevertheless M+1 and M+2 cannot be resolved by wide mass resolution (0.6 Da mass error). Consequently, for wide resolution, detected $^{18}O_1$ (M+2) contains M+1, M+2 or even M+3, corresponding to ca. 12% of total 1,5-InsP_8 or ca. 11% of total 5-InsP_7. The ratio of isotopic species found by qTOF is matched by this data.

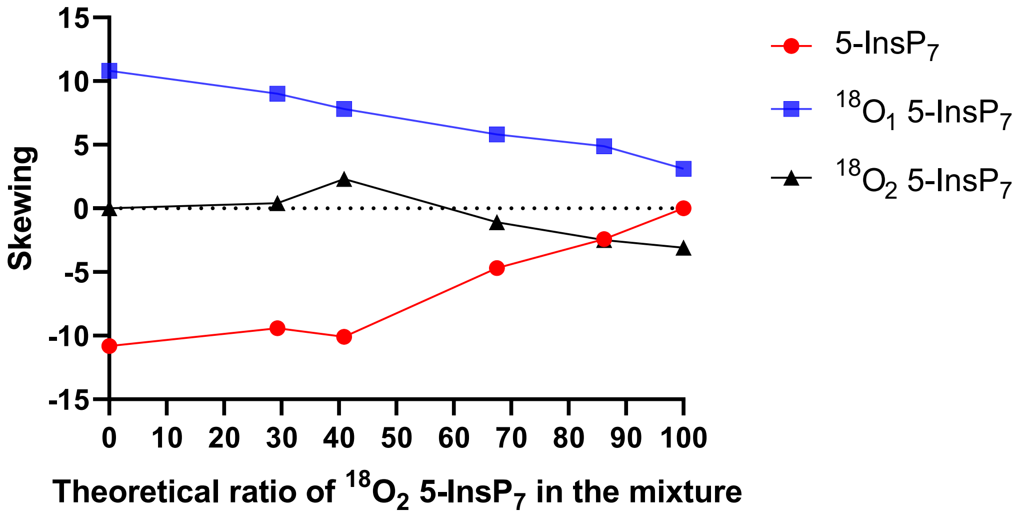

Supplementary Figure S13. Mixtures of synthetic $^{18}O_2$ 5-InsP$_7$ and unlabeled 5-InsP$_7$ with different compositions according to the theoretical ratio were prepared and analyzed by CE-QQQ. Skewing = experimental ratio (by CE-QQQ) - theoretical ratio.

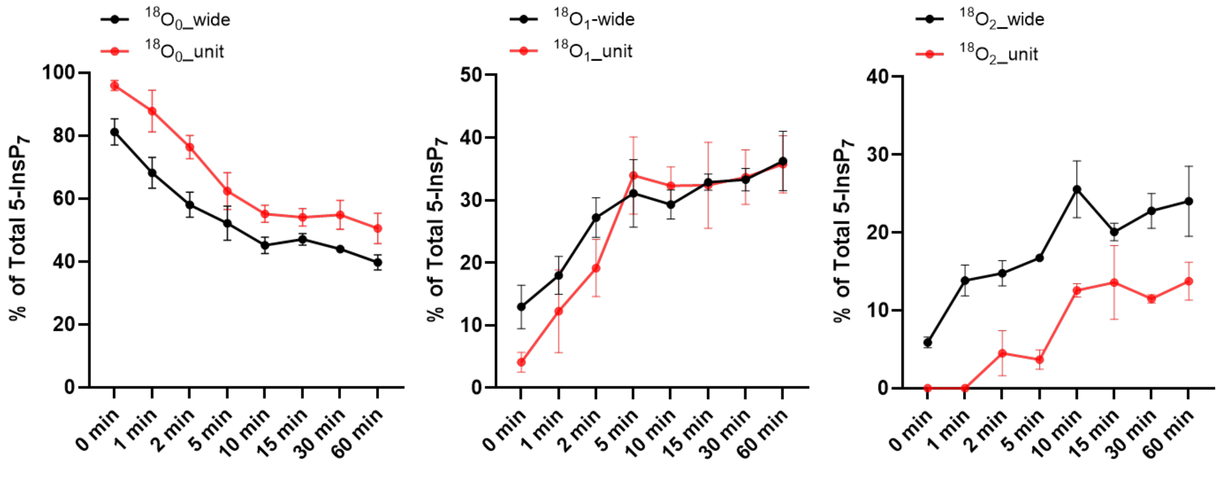

Supplementary Figure S14. 50% ^{18}O labeled water experiment: time-dependent formation of 5-InsP$_7$ isotopologues in yeast with different numbers of ^{18}O atoms studied by CE-QQQ wide mass resolution method and unit resolution method, respectively. The means of 3 samples with standard deviation are shown. Wild type yeast cells were grown logarithmically in SC medium at 20˚C. At the 0 min time point, the medium was changed to SC medium prepared with 50% of ^{18}O-labelled water (99% enrichment).

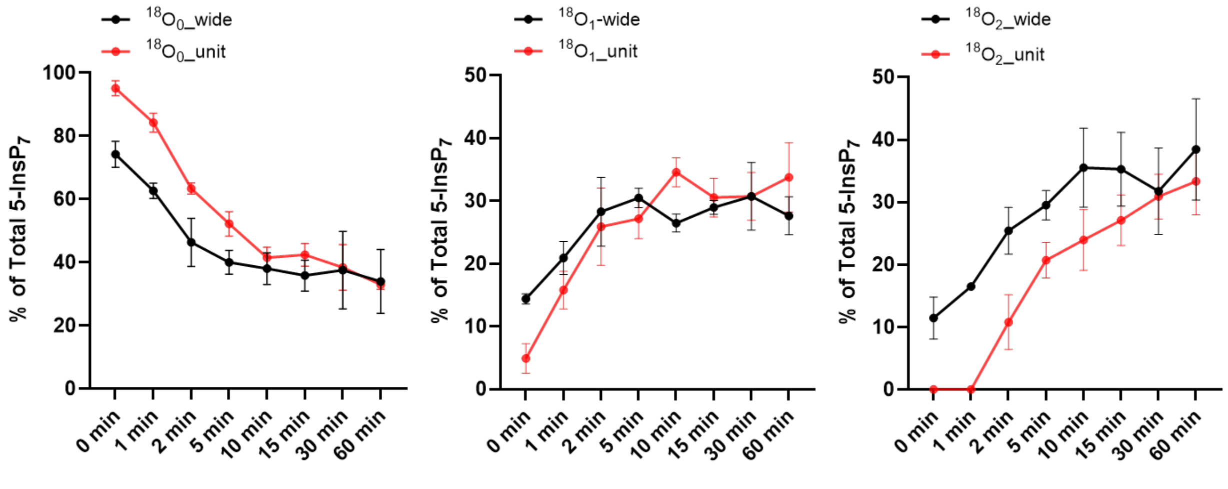

Supplementary Figure S15. 100% ^{18}O labeled water experiment: time-dependent formation of 5-InsP$_7$ isotopologues in yeast with different numbers of ^{18}O atoms studied by CE-QQQ wide mass resolution method and unit resolution method, respectively. The means of 3 samples with standard deviation are shown. Wild type yeast cells were grown logarithmically in SC medium at 20˚C. At the 0 min time point, the medium was changed to SC medium prepared with 100% of ^{18}O-labelled water (99% enrichment).

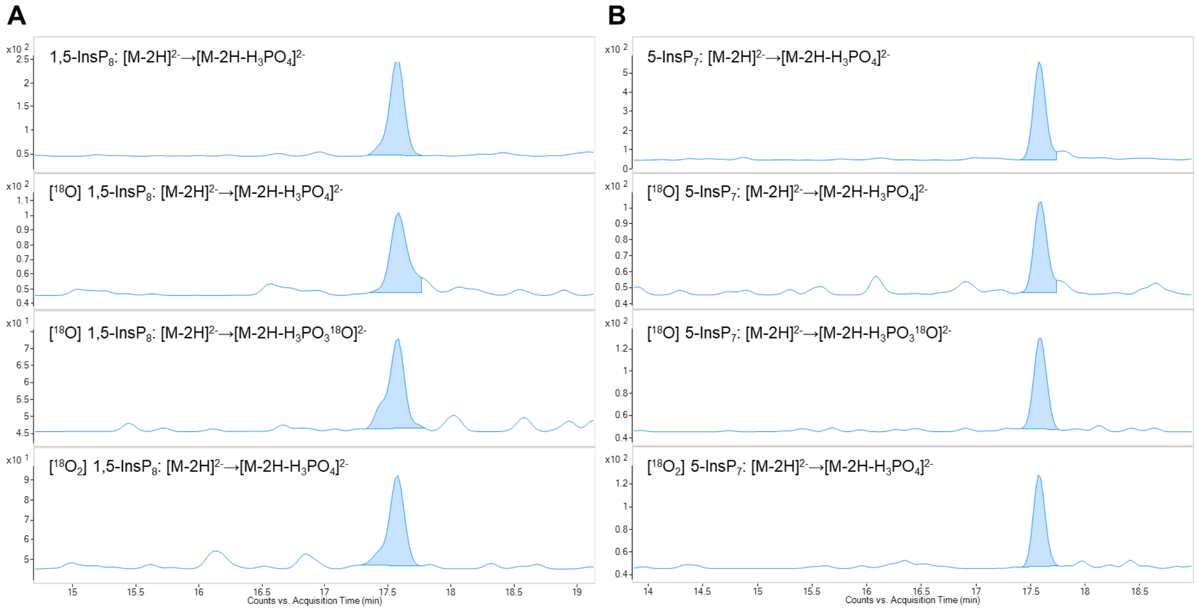

Supplementary Figure S16. Extracted ion electropherograms (EIEs) of unlabeled and ^{18}O labeled 1,5-InsP_8 (**A**) and 5-InsP_7 (**B**) from yeast under steady state conditions at the 1 min time point.

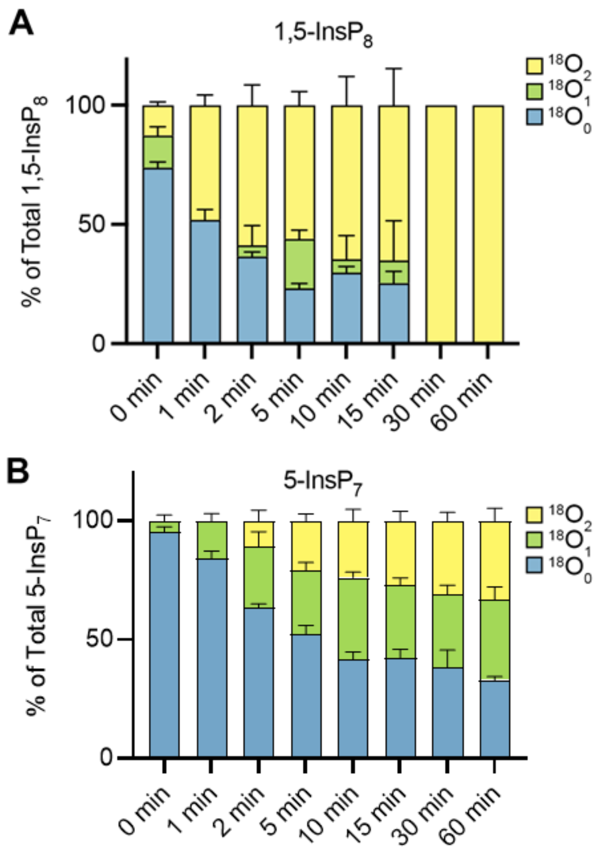

Supplementary Figure S17. Kinetics of ^{18}O entry into soluble InsPs of yeast under steady state conditions. Wild type cells were grown logarithmically in SC medium. The medium was changed to SC medium prepared with 100% of ^{18}O-labelled water. After further incubation at 20˚C for the indicated time periods, cells were extracted with perchloric acid. The means of triplicates are shown with standard deviation, representing: **A**. 1,5-$InsP_8$ (wide mass resolution), **B**. 5-$InsP_7$ (unit mass resolution).

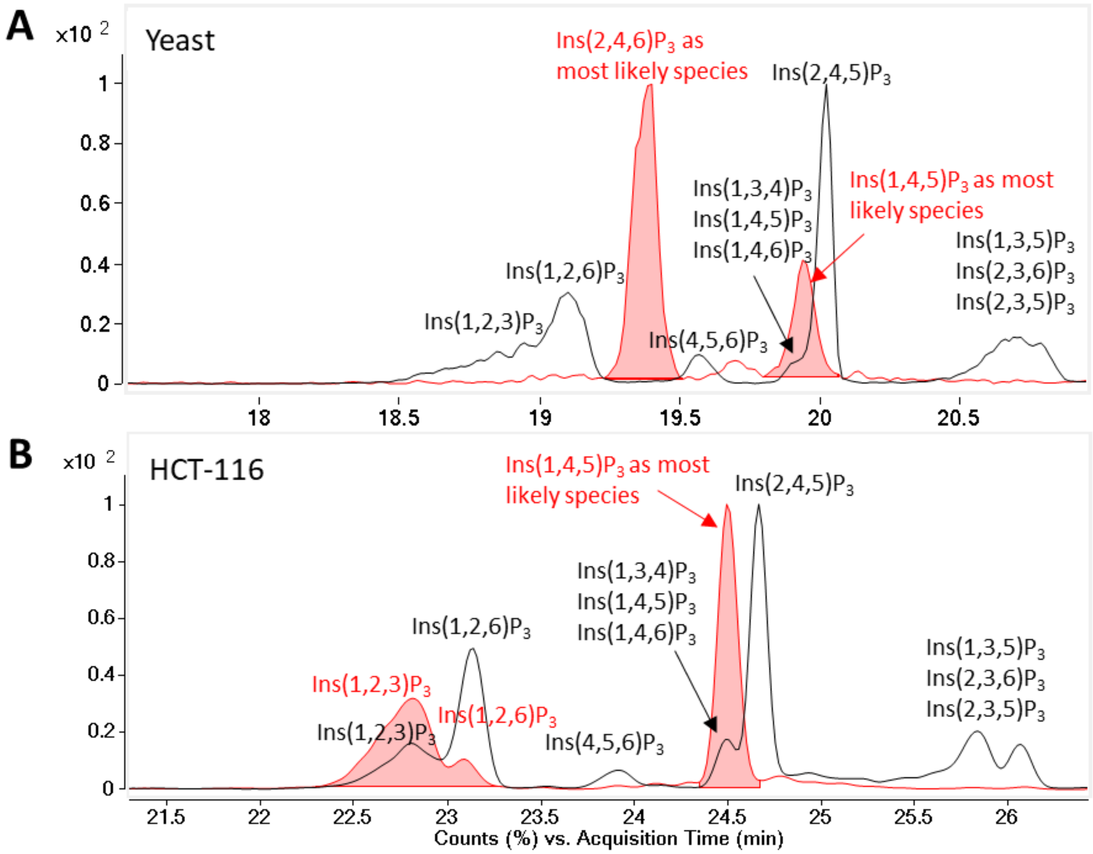

Supplementary Figure S18 Assignment of $InsP_3$ isomers in yeast and HCT-116 cells. **A** Extracted ion electropherograms of [$^{13}C_6$] $InsP_3$ reference (black line) and two $InsP_3$ in yeast (red area). The [$^{13}C_6$] $InsP_3$ reference were generated by incubating [$^{13}C_6$] $InsP_6$ (prepared with ultrapure water) at 100°C for 5 h. The assignment of each $InsP_3$ isomers were achieved as previously described (Liu *et al*, 2023). The second $InsP_3$ peak co-migrates with [$^{13}C_6$] Ins(1,3,4)P_3, Ins(1,4,5)P_3, Ins(1,4,6)P_3 and/or its enantiomers, and we infer that it is Ins(1,4,5)P_3 as most likely species. The references appear as part of a small shoulder before the Ins(2,4,5)P_3 peak, which is much more intense. **B** Extracted ion electropherograms of [$^{13}C_6$] $InsP_3$ reference (black line) and three $InsP_3$ species in HCT-116 cells (red area). The first $InsP_3$ peak co-migrates with [$^{13}C_6$] Ins(1,2,3)P_3 and the second $InsP_3$ peak co-migrates with [$^{13}C_6$] Ins(1,2,6)P_3 and/or its enantiomers. The third $InsP_3$ peak co-migrates with [$^{13}C_6$] Ins(1,3,4)P_3, Ins(1,4,5)P_3, Ins(1,4,6)P_3 and/or its enantiomers, and we infer that it is Ins(1,4,5)P_3 as most likely species.

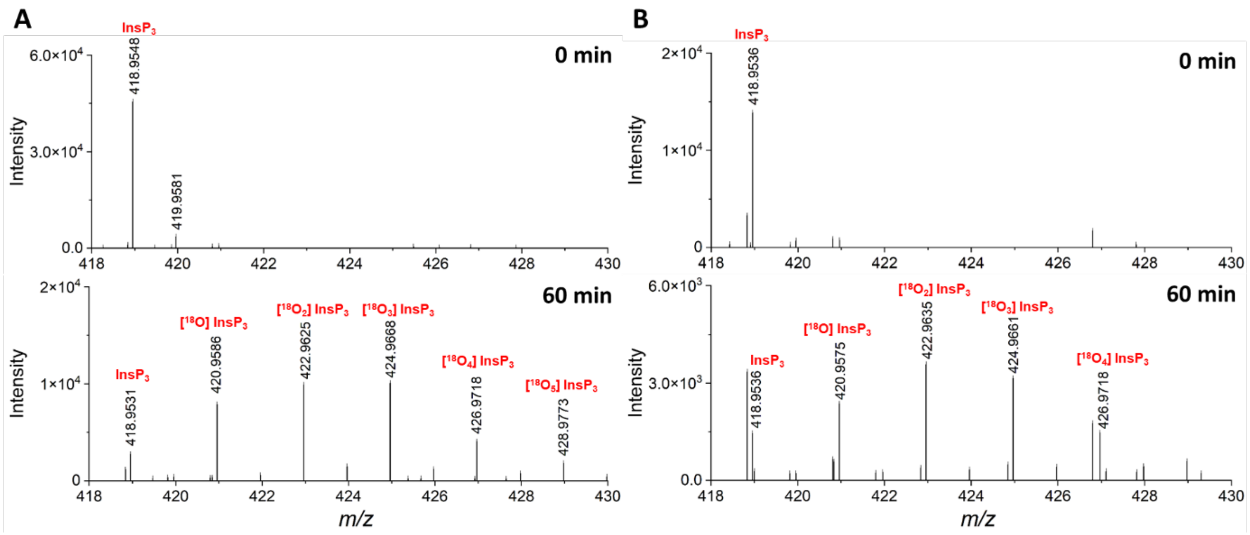

Supplementary Figure S19 qTOF analysis of $InsP_3$ in yeast. The analysis reveals the kinetics of ^{18}O incorporation into unassigned $InsP_3$-1 (**A**) and Ins(1,4,5)P_3 (**B**) at 0 min and 60 min time point. Theoretical $[M\text{-}H]^-$ for $InsP_3$, [^{18}O] $InsP_3$, [$^{18}O_2$] $InsP_3$, [$^{18}O_3$] $InsP_3$, [$^{18}O_4$] $InsP_3$, and [$^{18}O_5$] $InsP_3$ is 418.9551, 420.9593, 422.9636, 424.9678, 426.9721, 428.9763, respectively.

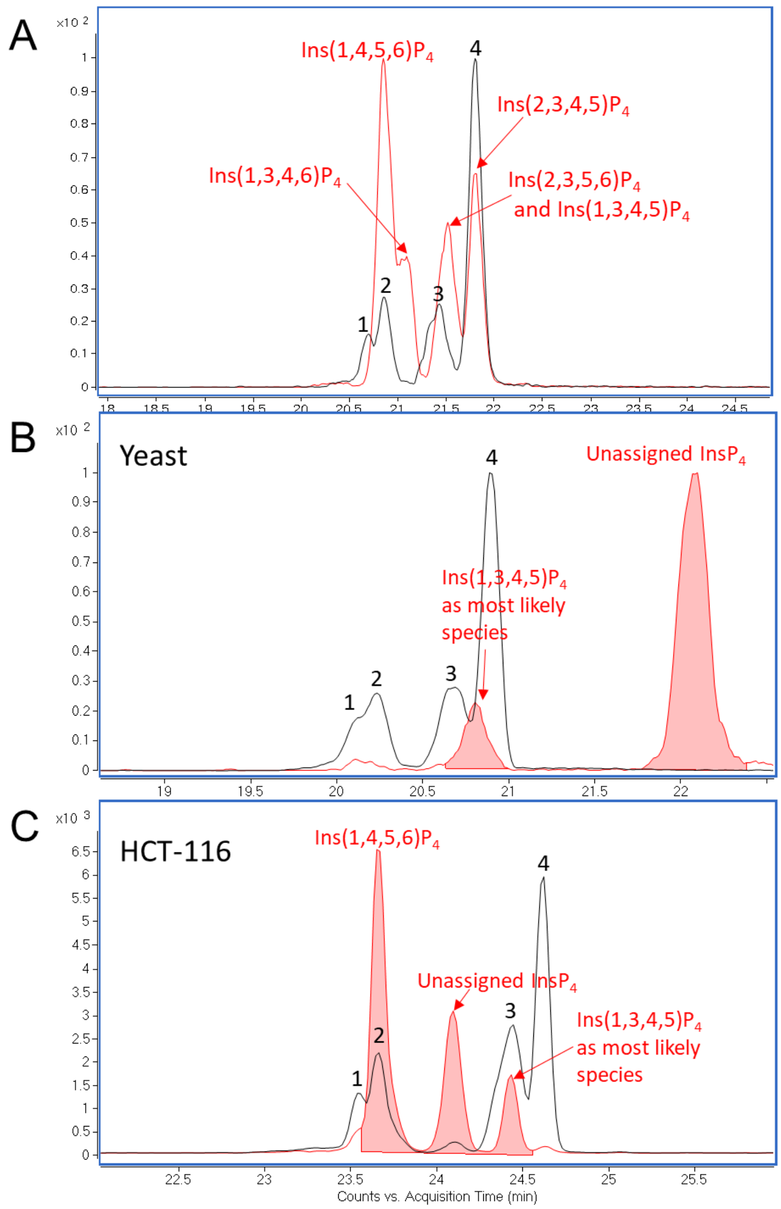

Supplementary Figure S20 Assignment of $InsP_4$ isomers in yeast and HCT-116 cells. **A** Extracted ion electropherograms of [$^{13}C_6$] $InsP_4$ reference (black line) and spiked-in commercial available $InsP_4$ standards (red line). The [$^{13}C_6$] $InsP_4$ reference were generated by incubating [$^{13}C_6$] $InsP_6$ (prepared with ultrapure water) at 100°C for 5 h. By comparing the migration time of [$^{13}C_6$] $InsP_4$ (black line) and $InsP_4$ references (red line), we assigned the [$^{13}C_6$] $InsP_4$ peak 2 as [$^{13}C_6$] Ins(1,4,5,6)P_4 and [$^{13}C_6$] $InsP_4$ peak 4 as [$^{13}C_6$] Ins(2,3,4,5)P_4. The peak of Ins(2,3,5,6)P_4 and Ins(1,3,4,5)P_4 overlapped and migrated between [$^{13}C_6$] $InsP_4$ peak 3 and [$^{13}C_6$] $InsP_4$ peak 4. However, given the

presence of 15 possible $InsP_4$ isomers, we cannot rule out other $InsP_4$ structural assignments. **B** Extracted ion electropherograms of [$^{13}C_6$] $InsP_4$ reference (black line) alongside two $InsP_4$ species detected in yeast (red area). The first $InsP_4$ species migrates between [$^{13}C_6$] $InsP_4$ peak 3 and 4, and we infer that it is Ins(1,3,4,5)P_4 as most likely species. The second $InsP_4$ species does not co-migrate with any $InsP_4$ isomer references presented in **A**. **C** Extracted ion electropherograms of [$^{13}C_6$] $InsP_4$ reference (black line) and three detected $InsP_4$ species in HCT-116 cells (red area). The first $InsP_4$ species co-migrates with [$^{13}C_6$] $InsP_4$ peak 2 which was assigned to [$^{13}C_6$] Ins(1,4,5,6)P_4. The second $InsP_4$ species does not co-migrate with any InsP4 references presented in **A**. The third $InsP_4$ species migrates between [$^{13}C_6$] $InsP_4$ peak 3 and 4, and we infer that it is Ins(1,3,4,5)P_4 as most likely species.

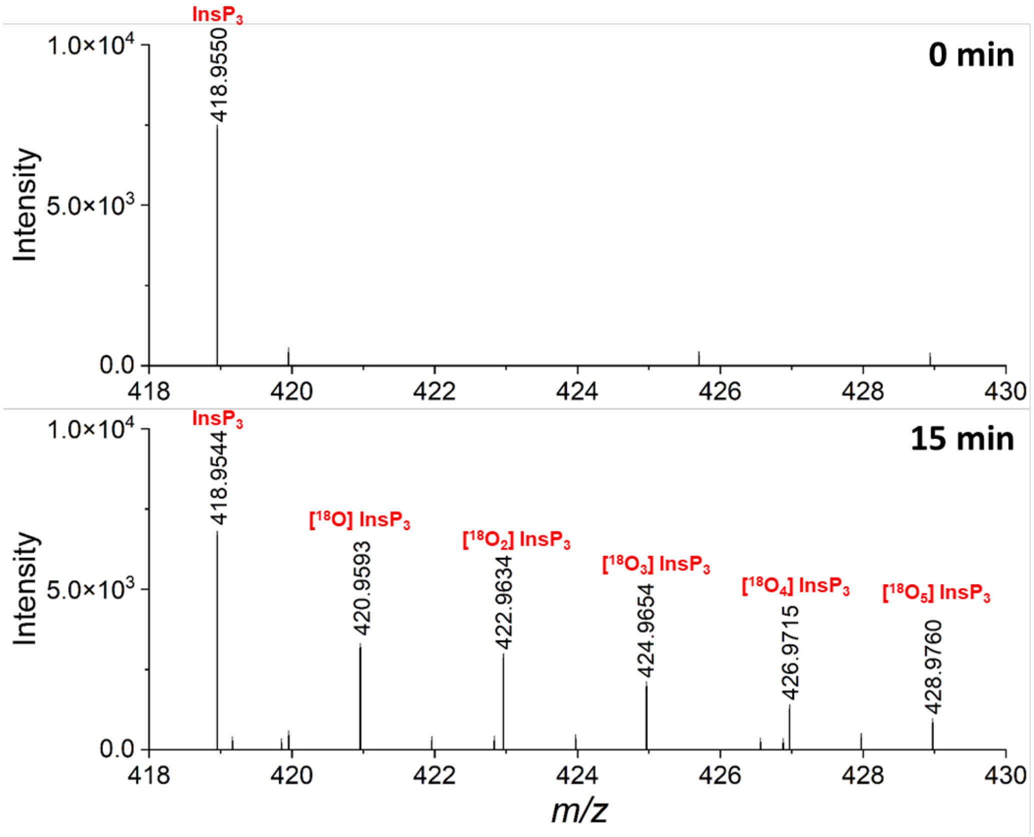

Supplementary Figure S21 qTOF analysis of Ins(1,4,5)P_3 in HCT-116 cells. The analysis reveals the kinetics of ^{18}O incorporation into Ins(1,4,5)P_3 at 0 min and 15 min time point. Theoretical $[M-H]^-$ for $InsP_3$, [^{18}O] $InsP_3$, [$^{18}O_2$] $InsP_3$, [$^{18}O_3$] $InsP_3$, [$^{18}O_4$] $InsP_3$, and [$^{18}O_5$] $InsP_3$ is 418.9551, 420.9593, 422.9636, 424.9678, 426.9721, 428.9763, respectively.

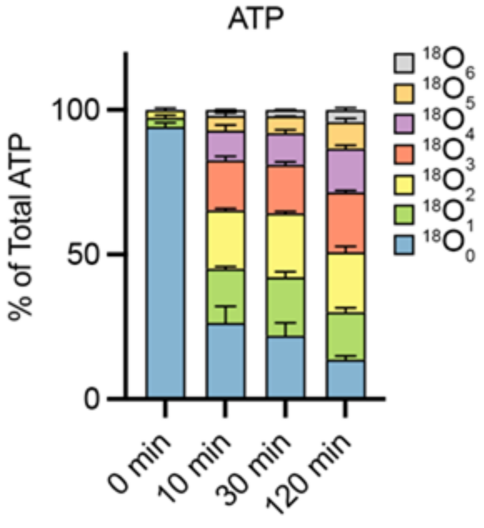

Supplementary Figure S22. Kinetics of ^{18}O entry into ATP from *D. discoideum*. Cells were grown in SIH medium, transferred to SIH media made of 50% of ^{18}O-labeled water. After further incubation for the indicated periods of time, samples were harvested and extracted. The means of two replicates with deviations are shown. CE-QQQ with wide mass resolution is applied.

Reference

Liu G, Riemer E, Schneider R, Cabuzu D, Bonny O, Wagner CA, Qiu D, Saiardi A, Strauss A, Lahaye T, et al (2023) The phytase RipBL1 enables the assignment of a specific inositol phosphate isomer as a structural component of human kidney stones. RSC Chem Biol 4: 300–309
